# Within- and between-host evolutionary effects on viral oncogenicity

**DOI:** 10.1093/ve/veaf043

**Published:** 2025-06-06

**Authors:** Yoshiki Koizumi, Michael B Bonsall

**Affiliations:** Department of Biology, University of Oxford, South Parks Road, Oxford OX1 3RB, United Kingdom; AIDS Clinical Center, National Center for Global Health and Medicine, Japan Institute for Health Security, 1-21-1 Toyama, Shinjuku-ku, Tokyo 162-8655, Japan; Department of Biology, University of Oxford, South Parks Road, Oxford OX1 3RB, United Kingdom

**Keywords:** oncovirus, virus dynamics, mathematical modelling, apparent competition, fitness landscape, nested model

## Abstract

Cancer-inducing viruses (oncogenic viruses) are linked to over 10% of cancer cases. Although the molecular details of viral oncogenesis are well-documented, the evolutionary mechanisms by which viruses have acquired oncogenic properties remain poorly understood. Here, we investigate the evolutionary conditions affecting viral oncogenicity across both within- and between-host scales using mathematical models of oncovirus–immune system interactions, conceptualized as an extended shared enemy–victim relationship. We begin by examining how oncogenic traits impact within-host viral dynamics, focusing on the transformation rate of infected cells into pre-cancerous states and the pre-cancerous cell proliferation rate. In various scenarios reflecting different within-host conditions, we then identify the transformation and proliferation rates that maximize within- and between-host viral fitness. We find that the transformation rate maximizing the viral load depends on the viral production rate, immunogenicity, and the immune-mediated elimination rate of pre-cancerous cells. We also identify conditions under which an intermediate proliferation rate minimizes within- and between-host viral fitness: in that scenario, a lower or higher proliferation rate leads to a higher viral load, providing a possible explanation for the diversity of oncogenic viruses. The analyses presented here provide insights into the evolutionary drivers affecting viral oncogenicity and highlight the complexity of oncogenic virus–immune system interactions.

## Introduction

Since the initial discovery of oncogenic viruses in the early twentieth century, extensive research has elucidated the mechanisms driving virus-induced oncogenesis (Javier and Butel 2008). A recent study showed that ~ 2.2 million new cancer cases worldwide are due to carcinogenic infections, with nearly 63% of these cases caused by oncogenic viruses (de Martel et al. 2020), such as human papillomavirus (HPV), hepatitis B virus (HBV), hepatitis C virus (HCV), Epstein–Barr virus (EBV), human herpesvirus type 8 (HHV-8, also known as Kaposi’s sarcoma herpesvirus, KSHV), Merkel cell polyomavirus (MCV), and human T-cell lymphotropic virus type-1 (HTLV-1). These viruses can exhibit common oncogenic traits, such as promoting cell proliferation, integrating viral genome into the cell genome, and evading immune surveillance by expressing their oncogenic proteins during both the viral productive and non-productive stages, increasing the risk of cancer development (Mesri et al. 2014). Some molecular examples underlying viral carcinogenicity include E7 oncoprotein in oncogenic HPVs (Munger et al. 1989), HBx oncoprotein in HBV (Tsai and Chung 2010), and Tax oncoprotein in HTLV-1 (Kehn et al. 2005), all of which share common targets in tumour suppressor pathways, leading to uncontrolled cell proliferation (Moore and Chang 2010). The capacity of these evolutionarily distinct viral oncoproteins to target similar intracellular proteins represents a case of convergent evolution (Moore and Chang 2010). Results from these previous studies have enhanced our understanding of the molecular mechanisms behind viral oncogenicity.

In contrast to the extensive research on the molecular mechanisms of viral oncogenicity, theoretical studies have expanded our insight by focusing on the evolutionary aspects of viral oncogenicity. In the context of virulence evolution, Murall et al. (2015) used mathematical models to investigate oncogenic HPVs dynamics at the within- and between-host levels. Theoretically, they validated a model in which vaccination against oncogenic HPVs modifies transmission dynamics, showing a scenario where a high proliferation rate of infected cells (driven by oncogene expression) allows transmission to occur before the host immune response clears the infection (Murall et al. 2015). Furthermore, Murall and Alizon (2019) compared the within-host life cycles of various oncogenic viruses to simulate the stochastic emergence of cancer cells from infected cells and assessed the oncogenic effects on virus fitness. These studies shed light on the evolutionary aspects of viral oncogenicity. Moreover, further research on within-host virus dynamics is needed to understand the evolutionary conditions that influence viral oncogenicity.

**Figure 1:**
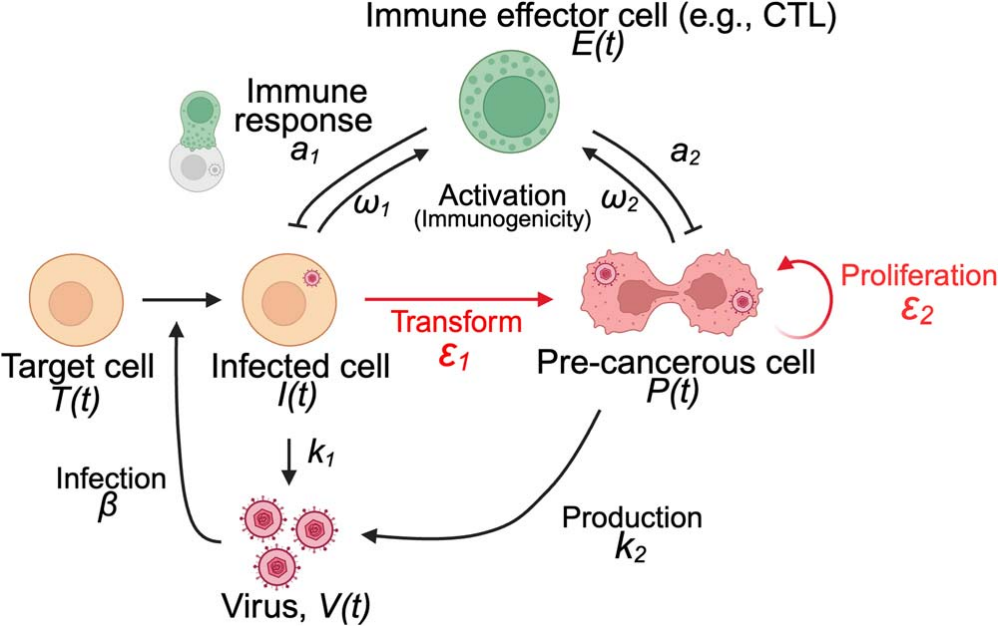
Schematic diagram of oncogenic virus infection and immune response. Target cells $T(t)$ are infected with oncogenic viruses $V(t)$ at rate $\beta$, and infected cells $I(t)$ transform into pre-cancerous cells $P(t)$ at rate ${\varepsilon}_1$. Infected cells produce progeny virions at rate ${k}_1$ and pre-cancerous cells produce virions at rate ${k}_2$. Pre-cancerous cells proliferate at rate ${\varepsilon}_2$. Immune effector cells $E(t)$ respond to these virion-producing cells at rates ${\omega}_1$ and ${\omega}_2$, eliminating them at rates ${a}_1$ and ${a}_2$, respectively. The full mathematical model is explained in the main text. This figure was created in BioRender. Koizumi, Y. (2025) https://BioRender.com/ihice0r.

Building on theoretical methods developed in previous studies (Nowak and May 2000, Perelson 2002, Murall et al. 2015), we hypothesize that reinterpreting oncovirus–immune interactions as an extended shared enemy–victim relationship, an established concept in ecology, could yield new insights into the evolutionary dynamics of viral oncogenicity. When considering two cell types, the dynamics between cells infected by oncogenic viruses that are not yet cancerous and pre-cancerous cells affected by oncogenic viruses (Fig. 1) embodies a form of apparent competition (Holt 1977). In other words, there is a relationship in which the two types of prey (in this case, infected cells with and without oncogenic effects) negatively affect each other through a shared predator (i.e. immune effector cells). The concept of apparent competition between two prey populations and a common predator has been explored extensively (Holt and Bonsall 2017). It offers a fundamental framework for enhancing our understanding of how the immune system influences pathogen diversity and the evolution of virulence within hosts (Cressler et al. 2016; Holt and Bonsall 2017). By investigating oncogenic viral dynamics in the context of apparent competition theory, we can gain insights into the evolutionary conditions that allow oncogenic viruses to persist, thereby applying ecological theory to the complex interactions of host–pathogen dynamics.

Here, we aim to improve understanding of the conditions that drive the within- and between-host evolution of viral oncogenicity by developing mathematical models of the oncovirus–immune system relationship. We evaluate the effects of varying viral oncogenicity, characterized by the transformation rate of infected cells to pre-cancerous states and the proliferation rate of pre-cancerous cells, on viral dynamics. We also explore the impacts of variations in viral production rate, immunogenicity, and immune-mediated elimination rate of pre-cancerous cells on the oncogenic parameters that maximize virus fitness. We thereby use ecological and epidemiological modelling to investigate the conditions favouring viral oncogenicity.

## Methods

### (a) Model of within-host oncogenic virus dynamics

We developed a mathematical model representing the interaction between the oncogenic virus and the host’s immune system (Fig. 1). Building upon the framework of a previous model (Murall et al. 2015), the temporal dynamics of the virus–host interaction are described by the following equations:


(1) \begin{equation*} \left.\begin{array}{l}\frac{dT(t)}{dt}=\lambda -\delta T(t)-\beta V(t)T(t),\\[3pt] {}\frac{dI(t)}{dt}=\beta V(t)T(t)-{\varepsilon}_1I(t)-{a}_1I(t)E(t)-\delta I(t),\\[3pt] {}\frac{dP(t)}{dt}={\varepsilon}_1I(t)+{\varepsilon}_2P(t)-{a}_2P(t)E(t)-\delta P(t),\\[3pt] {}\frac{dV(t)}{dt}={k}_1I(t)+{k}_2P(t)- cV(t),\\[3pt] {}\frac{dE(t)}{dt}={\omega}_1I(t)E(t)+{\omega}_2P(t)E(t).\end{array}\right\} \end{equation*}

Here, the state variables $T(t)$, $I(t)$, $P(t)$, $V(t)$ and $E(t)$ represent the populations of target uninfected cells, infected cells, pre-cancerous cells, virus, and immune effector cells, respectively. Uninfected cells are supplied at rate $\lambda$ and die at rate $\delta$, so that the maximum number of uninfected cells becomes $\lambda /\delta$ at a steady state in the absence of viral infection [i.e. $T(0)=\lambda /\delta$], which serves as a carrying capacity for uninfected cells. As it remains unclear whether the death rate of infected cells increases or decreases due to either the cytopathic effects of viruses or the immortalization of infected cells by viral oncogene expression, we assumed that infected and pre-cancerous cells die at the same rate $\delta$ to focus on the effects of oncogene-driven cell proliferation. The rate at which target cells become infected is governed by the parameter $\beta$, and the rate of viral clearance is governed by the parameter $c$. The transformation of infected cells into pre-cancerous cells occurs at rate ${\varepsilon}_1$.

Pre-cancerous cells are defined as infected cells that exhibit oncogenic effects such as increased cell proliferation and can be eliminated by the immune system, representing a stage preceding fully cancerous cells that the immune system cannot eliminate. Pre-cancerous cells proliferate exponentially at rate ${\varepsilon}_2$ without a carrying capacity, reflecting the assumption that oncogene expression induces unregulated cell divisions. To focus on the effect of oncogene-driven cell proliferation, we did not explicitly represent the proliferation term of infected cells [$I(t)$], as it is mathematically equivalent to adjusting the value of $\delta$ for $I(t)$. Although generalizing the properties of oncogenic effects sacrifices some aspects of biological realism, it allows us to examine how each parameter influences the life cycles of oncogenic viruses. Thus, our model assumes that the oncogenic potential of pre-cancerous cells is primarily their proliferation proficiency without differential mortality, and that each parameter of the model is independent, with no interactions between them.

Our model is distinct from previous approaches in several ways (Murall et al. 2015). First, we assume that the immune response is induced by infected and pre-cancerous cells at different rates (governed by the parameters ${\omega}_1$ and ${\omega}_2$, respectively). We assume that pre-cancerous cells originate from a single oncogenic virus strain and the immunogenicity of pre-cancerous cells (${\omega}_2$) is homogeneous for all cells. Second, we consider different immune-mediated elimination rates of infected (${a}_1$) and pre-cancerous cells (${a}_2$). Although there are many variations of mathematical models describing the immune responses to viruses (Wodarz 2007) and cancer (Mahlbacher et al. 2019), we select a simple, widely used model in which infected and pre-cancerous cells are eliminated by immune effector cells according to the principle of mass action. To capture viral clearance, we assumed that the activation rate of immune effector cells without a carrying capacity is higher than their decay rate and did not explicitly include the decay rate of immune effector cells or immune exhaustion during infection. Finally, the viral production rates of infected and pre-cancerous cells are specified by ${k}_1$ and ${k}_2$, respectively, accounting for the processes of virion release when cells die (i.e. ${k}_i$ is defined as the product of the death rate of infected cells and the viral burst size). These adaptations are intended to reflect the divergence in immunogenicity, immune-mediated elimination, and viral production rates between infected and pre-cancerous cells, which affect virus fitness.

As the life cycles of oncogenic viruses are highly diverse, it is difficult for a single, general model to capture all of the different features. However, to investigate important differences between these life cycles, we also analysed other models tailored to representative oncogenic viruses, such as the small DNA viruses (e.g. oncogenic HPVs) and the large DNA viruses (e.g. KSHV and EBV). In particular, we adapted a previous HPV-specific model developed by Murall et al. (2015) to our analysis [see Supplementary Information Section (e)]. For the large DNA viruses, we modified the general model in Equation (1) to address the scenarios in which pre-cancerous cells $P(t)$ do not produce virions and revert to virion-producing infected cells $I(t)$ [see Supplementary Information Section (c)].

### (b) Within-host virus fitness: total viral load, ${\boldsymbol{V}}_{\boldsymbol{total}}\left({\boldsymbol{\varepsilon}}_{\mathbf{1}},{\boldsymbol{\varepsilon}}_{\mathbf{2}}\right)$

Similarly to previous research (Murall et al. 2015), to evaluate virus fitness within a host, we measured the area under the within-host viral load curve:


(2) \begin{equation*} {V}_{total}\left({\varepsilon}_1,{\varepsilon}_2\right)={\int}_0^{t_{end}}V\left({\varepsilon}_1,{\varepsilon}_2,t\right) dt. \end{equation*}

In this expression, we explicitly note the dependence of the temporal trajectory of $V(t)$ [obtained from the system of equations in Equation (1)] on the rate at which infected cells transform into pre-cancerous cells and the pre-cancerous cell proliferation rate. The upper limit of the integral, ${t}_{end}$, is chosen to reflect the end of each infection; the value of ${t}_{end}$ is set for each realization of the model according to the threshold where $V(t)$ falls below 0.1 virions per millilitre. Although the within-host virus fitness is usually defined as a growth rate of a virus within a host, here we used the total viral load [${V}_{total}\left({\varepsilon}_1,{\varepsilon}_2\right)$] as the measure of within-host viral fitness to reflect the entire course of viral infection under the assumption that a higher total viral load increases the probability of viral transmission between hosts, providing an intuitive connection to the between-host scale. We also calculated the contribution of pre-cancerous cells to total viral production to assess the primary source of viral production during infection [see Supplementary Information Section (f)].

The system of ordinary differential equations in Equation (1) was solved numerically using the NDSolve function in *Mathematica*. Parameter values were set within a biologically plausible range, derived from previous studies on HCV (Neumann et al. 1998, Rong et al. 2010, Guedj et al. 2013, Ke et al. 2015), HIV-1 (Ribeiro et al. 2002, Asquith et al. 2006), and oncogenic HPVs (Murall et al. 2015) (see Table 1). We also evaluated the HPV-specific model using the parameter set used in previous studies (Murall et al. 2015) [see Supplementary Information Section (e)]. Initial conditions were set to $T(0)=\lambda /\delta$ (the uninfected steady state), $I(0)=1$, $P(0)=0$, $V(0)=1$, and $E(0)=0.01$, reflecting the onset of infection.

**Table 1:** Parameter values for the model of oncogenic viruses

Parameter	Definition	Default value (units)	References
$\boldsymbol{\lambda}$	Production rate of target cell	${10}^6$ (cells・ml^−1^・day^−1^)	(Ke et al. 2015)
$\boldsymbol{\delta}$	Death rate of cells	$0.15$ (day^−1^)	(Neumann et al. 1998; Ke et al. 2015)
$\boldsymbol{\beta}$	Infection rate	$8.88\times{10}^{-8}$ (ml・day^−1^・virion^−1^)	(Rong et al. 2010; Ke et al. 2015)
$\boldsymbol{c}$	Viral clearance rate	$22.3$ (day^−1^)	(Guedj et al. 2013; Ke et al. 2015)
${\boldsymbol{k}}_{\mathbf{1}}\&{\boldsymbol{k}}_{\mathbf{2}}$	Viral production rate	$56.5$ (day^−1^・dead cell^−1^)	(Ke et al. 2015)
${\boldsymbol{\omega}}_{\mathbf{1}}\&{\boldsymbol{\omega}}_{\mathbf{2}}$	Immune activation rate	$0.001$ (day^−1^)	(Ribeiro et al. 2002; Murall et al. 2015)
${\boldsymbol{a}}_{\mathbf{1}}\&{\boldsymbol{a}}_{\mathbf{2}}$	Immune-mediated elimination rate	$0.01$ (day^−1^)	(Asquith et al. 2006; Murall et al. 2015)
${\boldsymbol{\varepsilon}}_{\mathbf{1}}$	Transformation rate	$1.0$ (day^−1^)	*
${\boldsymbol{\varepsilon}}_{\mathbf{2}}$	Proliferation rate	$1.0$ (day^−1^)	*
$\boldsymbol{\mu}$	Natural mortality rate of a host	${10}^{-4}$ (day^−1^)	**
$\boldsymbol{m}$	Additional mortality rate by infection	$1.0$ (cell^−1^)	**
$\boldsymbol{b}$	Scaling factor of viral transmission ($b={\beta}_{BH}{S}_0$)	$5.865\times{10}^{-5}$	***

^*^Conduct calculations across a range of values: 0.1–10.

^**^We fixed $\mu$ at ${10}^{-4}$ and performed calculations assuming baseline virulence at $m=1.0$, higher virulence at $m=5$, and lower virulence at $m=0.1$ (see Supplementary Information).

^***^ $b\ \left(={\beta}_{BH}{S}_0\right)$ is a scaling factor of viral transmission, and its value was adjusted such that the between-host reproduction number of the virus without oncogenic effects is 1 (i.e. $R\left(0,0\right)=1$).

### (c) Between-host virus fitness: reproduction number, $\boldsymbol{R}\left({\boldsymbol{\varepsilon}}_{\mathbf{1}},{\boldsymbol{\varepsilon}}_{\mathbf{2}}\right)$

To assess virus fitness at the between-host scale linked to the within-host viral dynamics, we used the framework from previous studies on virulence evolution (Gilchrist and Coombs 2006, Coombs et al. 2007). In this framework, the key measure of the between-host virus fitness is the basic reproduction ratio ${R}_0$, defined as the expected number of secondary infections from a single infected host during its infectious period in a fully susceptible population. While ${R}_0$ can be derived using various approaches (Heffernan et al. 2005, van den Driessche 2017), we used a standard method based on the susceptible-infected epidemiological model, which involves multiplying the host infectiousness by the survival function of the infected host:


(3) \begin{equation*} {R}_0={S}_0{\int}_0^{\infty }B(t)F(t) dt, \end{equation*}

where ${S}_0$ is the equilibrium density of susceptible hosts in a virus-free environment, $B(t)$ is the host infectiousness at time $t$, and $F(t)$ is the survival probability that an infected host remains infectious until time $t$. Here, we assumed the simple scenario where a single virus strain is introduced into a homogeneous host population and spreads by mass-action transmission, rather than considering more complex scenarios such as the heterogeneity of host populations, spatial structure, co- and super-infection, and host co-evolution. Similar to other work (Murall et al. 2015), we used the simple expression for $B(t)$ such that this host infectiousness is linearly proportional to the within-host viral load, expressed as $B(t)={\beta}_{BH}V\left({\varepsilon}_1,{\varepsilon}_2,t\right)$, where ${\beta}_{BH}$ is a constant for the host infectiousness. We also analysed a scenario in which $B(t)$ is a saturating function described by a Hill function of the within-host viral load [see Supplementary Information Section (d)]. Based on the definition of virulence caused by viral infection (Gilchrist and Coombs 2006, Coombs et al. 2007), we used the survival function $F(t)$ as follows:


(4) \begin{equation*} F(t)=\exp \left(-\mu t-\mu m{\int}_0^t\left(T(0)-T\left({\varepsilon}_1,{\varepsilon}_2,z\right)\right) dz\right), \end{equation*}

where $\mu$ is the natural mortality of uninfected hosts, and $m$ is the additional mortality due to the viral infection, defined as a constant, independent parameter distinct from other processes such as the transformation rate (${\varepsilon}_1$) and the proliferation rate (${\varepsilon}_2$). We characterized viral oncogenicity as a process by which infected cells transform and increase cellular proliferation, affecting within-host viral dynamics without changing the value of $m$. In other words, different values of $m$ reflect varying degrees of viral virulence in the absence of cancer development. We fixed $\mu ={10}^{-4}$ and performed calculations assuming the baseline virulence at $m=1.0$, higher virulence at $m=5.0$ and lower virulence at $m=0.1$ [see Supplementary Information Section (b)]. We assumed that the survival function $F(t)$ decreased exponentially in response to the consumption of host resources by viral infection [corresponding to $\mu m{\int}_0^t\left(T(0)-T\left({\varepsilon}_1,{\varepsilon}_2,z\right)\right) dz$ in Equation (4)], accounting for the decrease in host activity due to viral infection, which leads to a reduced infectious period.

To explore the effects of varying oncogenic parameters (${\varepsilon}_1$ and ${\varepsilon}_2$) on between-host virus fitness, we adapted the concept of ${R}_0$ into the between-host reproduction number $R\left({\varepsilon}_1,{\varepsilon}_2\right)$:


(5) \begin{eqnarray*}&& R\left({\varepsilon}_1,{\varepsilon}_2\right) \nonumber\\&& ={\int}_0^{t_{end}} bV\left({\varepsilon}_1,{\varepsilon}_2,t\right)\exp \left(-\mu t-\mu m{\int}_0^t\left(T(0)-T\left({\varepsilon}_1,{\varepsilon}_2,z\right)\right) dz\right) dt,\nonumber\\ \end{eqnarray*}

where $b={\beta}_{BH}{S}_0$ serves as a scaling factor of viral transmission, and $R\left({\varepsilon}_1,{\varepsilon}_2\right)$ is the total number of secondary infections caused by a single infected host during the infectious period from $t=0$ to ${t}_{end}$. The value of $b$ was set so that the between-host reproduction number of the virus without oncogenic effects is 1 [i.e. $R\left(0,0\right)=1$], which means that if $R\left({\varepsilon}_1,{\varepsilon}_2\right)>1$, the virus with oncogenic effects is more advantageous, whereas if $R\left({\varepsilon}_1,{\varepsilon}_2\right)<1$, the oncogenic effects do not provide an advantage to viruses. To predict the evolutionary consequences of viral oncogenicity under different conditions, we assume that viral evolution favours the direction of higher $R\left({\varepsilon}_1,{\varepsilon}_2\right)$. Although there are some limitations in using the between-host reproduction number as a measure of viral evolution (Lion and Metz 2018), this approach enables us to evaluate the fitness landscape for oncogenic viruses. We numerically calculated $R\left({\varepsilon}_1,{\varepsilon}_2\right)$ for various patterns of oncogenic parameters (${\varepsilon}_1$ and ${\varepsilon}_2$) using *Mathematica* and constructed the fitness landscape of $R\left({\varepsilon}_1,{\varepsilon}_2\right)$.

## Results

We began by assessing the impact of varying the oncogenic effects on viral dynamics at the within-host level. Specifically, we varied the transformation rate of infected cells to the pre-cancerous state (${\varepsilon}_1$) and the proliferation rate of pre-cancerous cells (${\varepsilon}_2$). We then investigated how the optimal values of these oncogenic parameters for within- and between-host virus fitness, ${V}_{total}$ and $R\left({\varepsilon}_1,{\varepsilon}_2\right)$, are influenced by the values of three key model factors: the viral production rates (${k}_1$ and ${k}_2$), immunogenicity (${\omega}_1$ and ${\omega}_2$), and the immune-mediated elimination rates (${a}_1$ and ${a}_2$).

### (a) Contributions of oncogenic effects to viral dynamics

**Figure 2:**
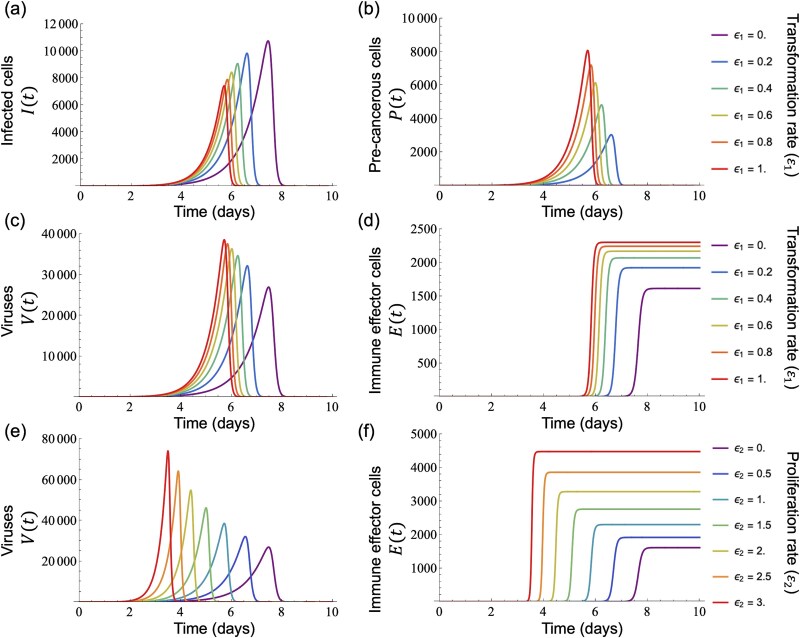
Simulations of oncogenic viral infection and immune response dynamics under varying oncogenic effects. Each coloured line represents a distinct simulation set, with brighter colours indicating higher oncogenic effect intensities. Panels (a)–(d) show the impacts of varying transformation rate ${\varepsilon}_1$ on (a) infected cells $I(t)$, (b) pre-cancerous cells $P(t)$, (c) within-host viral load $V(t)$, and (d) immune effector cells $E(t)$. A higher transformation rate ${\varepsilon}_1$ increases pre-cancerous cells and within-host viral load, with a faster and stronger immune response. Panels (e) and (f) show the effects of changing the proliferation rate ${\varepsilon}_2$ on (e) within-host viral load $V(t)$ and (f) immune effector cells $E(t)$, respectively. Increasing proliferation rate ${\varepsilon}_2$ results in a higher viral peak, promoting a faster and higher immune response.

To investigate the impact of oncogenic effects on viral dynamics and immune responses, we solved the system of equations in Equation (1) numerically with the parameter values from Table 1. In Fig. 2a–d, we varied the transformation rate (${\varepsilon}_1$), a key oncogenic effect, from 0 to 1. Consistent with findings from previous studies (Murall et al. 2015), a higher transformation rate (${\varepsilon}_1$) led to an increased number of pre-cancerous cells, which contributed to a higher peak viral load (Fig. 2b and c). Simultaneously, the induction of immune responses by virion-producing cells caused an early and substantial increase in the number of immune effector cells (Fig. 2d). Similarly, in Fig. 2e and f, increasing the proliferation rate (${\varepsilon}_2$) of pre-cancerous cells also resulted in a higher viral peak and an earlier immune response, following the same pattern observed with an increased transformation rate (${\varepsilon}_1$) (Fig. 2c and d). Overall, while higher oncogenic effects (${\varepsilon}_1$ and ${\varepsilon}_2$) increased the viral peak, rapid elimination by the immune system led to an earlier termination of the infection.

### (b) Individual effects of viral production rates, immunogenicity, and immune-mediated elimination rates on oncogenic outcomes

#### (1) Viral production rates

**Figure 3:**
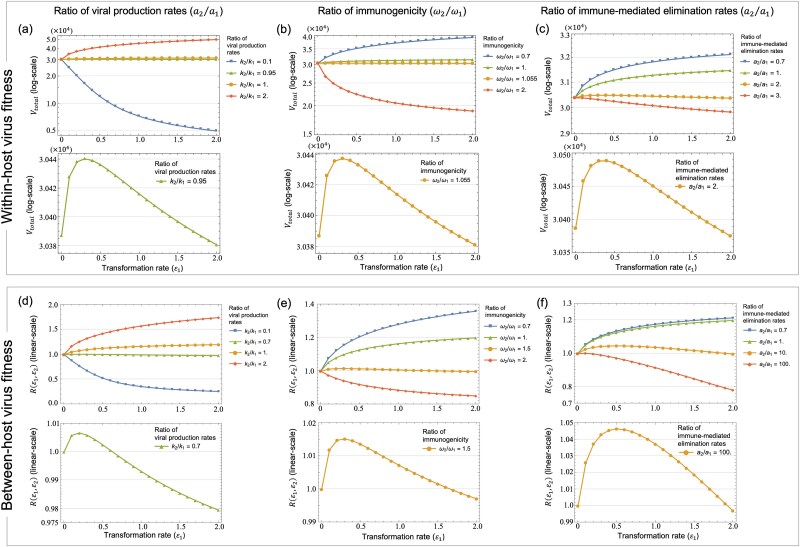
Effects of different transformation rates on the within- and between-host virus fitness, ${V}_{total}$ and $R\left({\varepsilon}_1,{\varepsilon}_2\right)$, respectively, with varying the ratios of viral production, immunogenicity, and immune response rate in infected cells and pre-cancerous cells. The horizontal axis is the transformation rate ${\varepsilon}_1$, and the vertical axis is the within-host total viral load ${V}_{total}$ in (a)–(c) and the between-host reproduction number $R\left({\varepsilon}_1,{\varepsilon}_2\right)$ in (d)–(f). The colour from blue to red indicates increasing the ratios of viral production rates ${k}_2/{k}_1$ in (a) and (d), immunogenicity ${\omega}_2/{\omega}_1$ in (b) and (e), and immune response rates ${a}_2/{a}_1$ in (c) and (f). These ratios were varied by fixing ${k}_1=56.5$, ${\omega}_1=0.001$, and ${a}_1=0.01$, and then varying ${k}_2$, ${\omega}_2$, and ${a}_2$, respectively. Other ratios were set to 1, and ${\varepsilon}_2$ was fixed at 1. The bottom panels in (a)–(f) show a zoomed view of the vertical axis, focusing on a specific line from the corresponding top panels [e.g. the bottom panel in (a) shows the result for ${k}_2/{k}_1=0.95$ (green) from the top panel in (a)]. Note that the bottom panels show the intermediate transformation rates that maximize ${V}_{total}$ in (a)–(c) and $R\left({\varepsilon}_1,{\varepsilon}_2\right)$ in (d)–(f).

**Figure 4:**
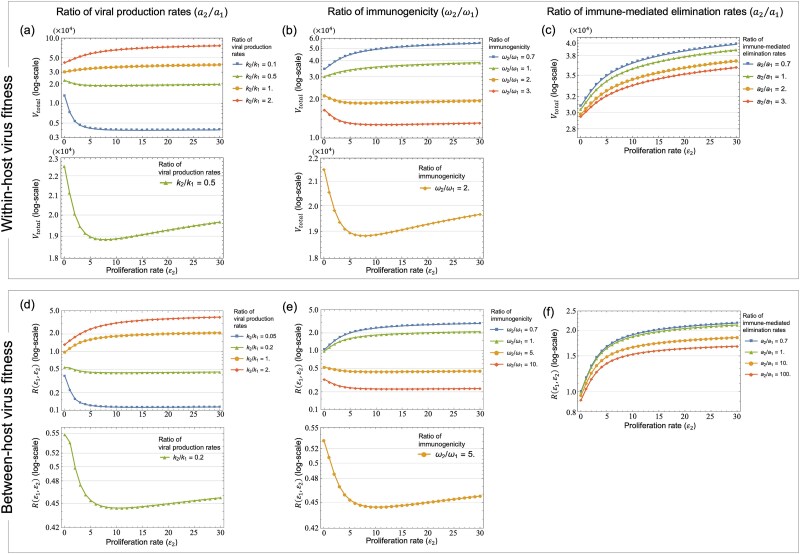
Influence of different proliferation rates on the within- and between-host virus fitness, ${V}_{total}$ and $R\left({\varepsilon}_1,{\varepsilon}_2\right)$, respectively, with varying the ratios of viral production, immunogenicity, and immune response rate in infected cells and pre-cancerous cells. The horizontal axis is the proliferation rate ${\varepsilon}_2$ of pre-cancerous cells, and the vertical axis is the within-host total viral load ${V}_{total}$ in (a)–(c) and the between-host reproduction number $R\left({\varepsilon}_1,{\varepsilon}_2\right)$ in (d)–(f). The colour from blue to red indicates increasing the ratios of viral production rates ${k}_2/{k}_1$ in (a) and (d), immunogenicity ${\omega}_2/{\omega}_1$ in (b) and (e), and immune response rates ${a}_2/{a}_1$ in (c) and (f). These ratios were varied by fixing ${k}_1=56.5$, ${\omega}_1=0.001$, and ${a}_1=0.01$, and then varying ${k}_2$, ${\omega}_2$, and ${a}_2$, respectively. Other ratios were set to 1, and ${\varepsilon}_1$ was fixed at 1. The bottom panels in (a), (b), (d), and (e) show a zoomed view of the vertical axis, focusing on a specific line from the corresponding top panels [e.g. the bottom panel in (a) shows the result for ${k}_2/{k}_1=0.5$ (green) from the top panel in (a)]. Note that the bottom panels show the intermediate proliferation rates that minimize ${V}_{total}$ in (a) and (b) and $R\left({\varepsilon}_1,{\varepsilon}_2\right)$ in (d) and (e). In (c) and (f), ${V}_{total}$ and $R\left({\varepsilon}_1,{\varepsilon}_2\right)$ increased consistently with ${\varepsilon}_2$, regardless of the ${a}_2/{a}_1$ ratios.

We examined the effects of variations in viral production ratio (${k}_2/{k}_1$) on the oncogenic advantage in terms of the within-host total viral load ${V}_{total}$ as the within-host virus fitness (Figs 3a and 4a) and the between-host reproduction number $R\left({\varepsilon}_1,{\varepsilon}_2\right)$ as the between-host virus fitness (Figs 3d and 4d). When viral production rates were equal in infected cells and pre-cancerous cells (${k}_2/{k}_1=1$, yellow lines in Figs 3a and d and 4a and d) or higher in pre-cancerous cells (${k}_2/{k}_1=2$, red lines in Figs 3a and d and 4a and d), increased transformation and proliferation rates, reflecting high oncogenicity, consistently led to higher within-host total viral load and between-host reproduction number. Conversely, with pre-cancerous cells producing fewer viral particles than infected cells (blue line: ${k}_2/{k}_1=0.1$ in Figs 3a and d and 4a, and ${k}_2/{k}_1=0.05$ in Fig. 4d), lower transformation and proliferation rates (i.e. low oncogenicity) led to higher within-host total viral load and between-host reproduction number. We note that a reduction in viral output from pre-cancerous cells (green line: ${k}_2/{k}_1=0.95$ in Fig. 3a and ${k}_2/{k}_1=0.7$ in Fig. 3d) leads to an intermediate optimal transformation rate for maximizing both the within-host total viral load and the between-host reproduction number, suggesting a trade-off between increased viral production and the risk of the elimination by the immune system due to higher transformation rates. Furthermore, with a more moderate decrease in viral production of pre-cancerous cells (green line: ${k}_2/{k}_1=0.5$ in Fig. 4a and ${k}_2/{k}_1=0.2$ in Fig. 4d), the within-host total viral load and the between-host reproduction number reached their minimum at intermediate proliferation rates of around ${\varepsilon}_2=8$ and ${\varepsilon}_2=10$, respectively, as the disadvantages of the elimination by the immune system surpassed the benefits of increased viral production by pre-cancerous cells with an intermediate proliferation rate. These results suggest a divergence in oncogenic strategies for maximizing virus fitness within and between hosts: when pre-cancerous cells produce fewer viral particles than infected cells, an intermediate transformation rate can maximize the within-host total viral load and the between-host reproduction number while an intermediate proliferation rate can minimize both.

#### (2) Immunogenicity

We then investigated the effect of variations in immune induction rates of each virion-producing cell, termed immunogenicity (${\omega}_1$ and ${\omega}_2$), on the within-host total viral load and the between-host reproduction number. Initially, when immunogenicity was equivalent in both cell types (${\omega}_2/{\omega}_1=1.0$), higher transformation and proliferation rates, i.e. high oncogenicity, resulted in higher within-host total viral load and between-host reproduction number (green lines in Figs 3b and e and 4b and e). When instead pre-cancerous cells were more immunogenic than infected cells (${\omega}_2/{\omega}_1=2.0$), increased oncogenicity was not beneficial for viral production: lower transformation rates maximized both the within-host total viral load and the between-host reproduction number (red line in Fig. 3b and e). Higher immunogenicity of pre-cancerous cells also indicated a fitness minimum for the proliferation rate of around ${\varepsilon}_2=8$ (yellow line in the bottom panel of Fig. 4b and e). Conversely, with immune evasion occurring in pre-cancerous cells (${\omega}_2/{\omega}_1=0.7$), higher transformation and proliferation rates were optimal in terms of maximizing both the within-host total viral load and the between-host reproduction number (blue lines in Figs 3b and e and 4b and e). In the case of slightly increased immunogenicity of pre-cancerous cells, an optimal transformation rate of ${\varepsilon}_1=0.3$ maximized both the total within-host viral load and the between-host reproduction number (yellow line: ${\omega}_2/{\omega}_1=1.055$ in the bottom panel of Fig. 3b and ${\omega}_2/{\omega}_1=1.5$ in the bottom panel of Fig. 3e).

#### (3) Immune-mediated elimination rates

We explored the influence of different immune-mediated elimination ratios (${a}_2/{a}_1$) on the within-host total viral load and the between-host reproduction number. With equal elimination rates in both cell types (${a}_2/{a}_1=1.0$), increased transformation and proliferation rates corresponded with a higher within-host total viral load (green lines in Figs 3c and f and 4c and f). More effective immune escape of pre-cancerous cells (${a}_2/{a}_1=0.7$) also favoured high oncogenicity to maximize both the within-host total viral load and the between-host reproduction number (blue lines in Figs 3c and f and 4c and f). In contrast, with pre-cancerous cells being more susceptible to immune-mediated elimination, lower transformation rates were most effective in maximizing both the within-host total viral load and the between-host reproduction number (red line: ${a}_2/{a}_1=3.0$ in Fig. 3c and ${a}_2/{a}_1=1000$ in Fig. 3f). Increased immune-mediated elimination in pre-cancerous cells (${a}_2/{a}_1=2.0$ in Fig. 3c and ${a}_2/{a}_1=100$ in Fig. 3f) indicated an optimal transformation rate around ${\varepsilon}_1=0.3\sim 0.5$ for maximizing the within-host total viral load and the between-host reproduction number, respectively (yellow line in the bottom panels of Fig. 3c and f). We note that a higher proliferation rate consistently maximized both the within-host total viral load and the between-host reproduction number across different ${a}_2/{a}_1$ ratios (Fig. 4c and f), suggesting that the increased proliferation of pre-cancerous cells compensates for the immune-mediated elimination of virion-producing cells.

### (c) Systematic analysis of combined immune response variations on virus fitness in oncogenic contexts

**Figure 5:**
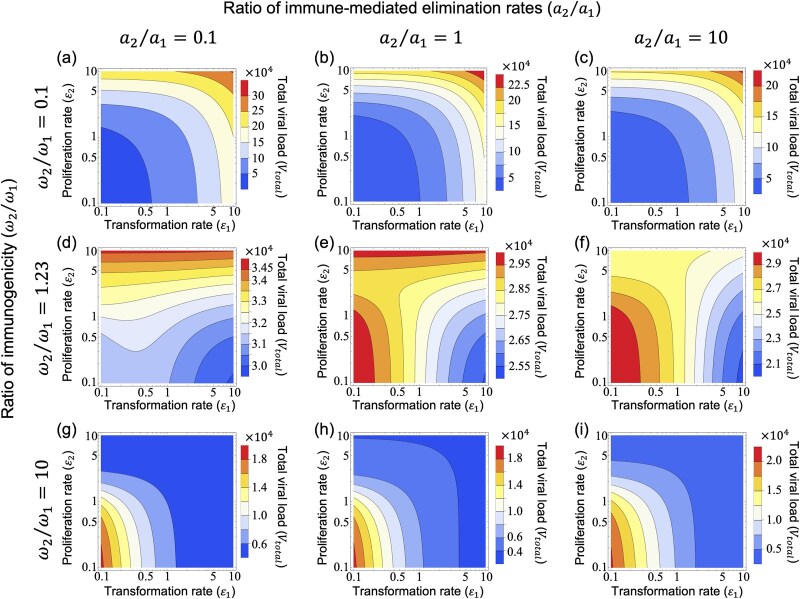
Effects of different transformation and proliferation rates on the within-host total viral load depending on immunogenicity and immune response in infected cells and pre-cancerous cells. Contour maps showing the changes in within-host total viral load (${V}_{total}$) in response to various combinations of immunogenicity (${\omega}_2/{\omega}_1$) and immune-mediated elimination rates (${a}_2/{a}_1$), plotted against transformation rates (${\varepsilon}_1$, x-axis, log-scale) and proliferation rates (${\varepsilon}_2$, y-axis, log-scale). From top to bottom, the rows increase the ratios of immunogenicity (${\omega}_2/{\omega}_1=0.1,1.23,10$), and the columns, from left to right, increase the ratios of immune-mediated elimination rates (${a}_2/{a}_1=0.1,1,10$), with fixed ${\omega}_1=0.001$, ${a}_1=0.01$, and the viral production ratio (${k}_2/{k}_1=1$). The colour transition from blue to red indicates increasing ${V}_{total}$ values. Panels (a)–(c) show an increase in ${V}_{total}$ towards the upper-right corner at ${\omega}_2/{\omega}_1=0.1$. Panels (g)–(i) show an opposite trend with ${V}_{total}$ increasing towards the lower-left corner at ${\omega}_2/{\omega}_1=10$. Panels (d)–(f) show a transitional pattern at ${\omega}_2/{\omega}_1=1.23$. Note that (e) displays a distinctive dual-peak pattern in ${V}_{total}$, located in the upper and lower-left regions.

**Figure 6:**
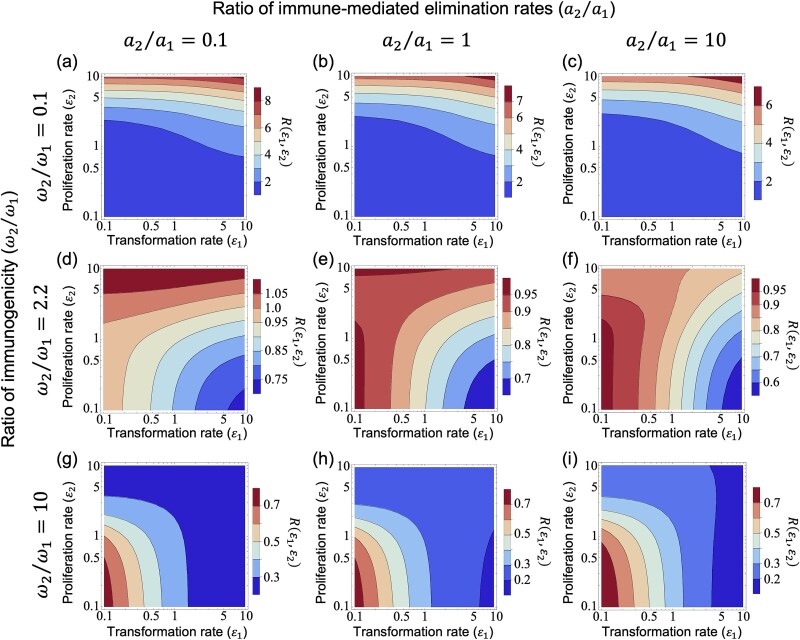
Effects of different transformation and proliferation rates on the between-host virus fitness $R\left({\varepsilon}_1,{\varepsilon}_2\right)$ depending on immunogenicity and immune response in infected cells and pre-cancerous cells. Contour maps showing the changes in $R\left({\varepsilon}_1,{\varepsilon}_2\right)$ in response to various combinations of immunogenicity (${\omega}_2/{\omega}_1$) and immune-mediated elimination rates (${a}_2/{a}_1$), plotted against transformation rates (${\varepsilon}_1$, x-axis, log-scale) and proliferation rates (${\varepsilon}_2$, y-axis, log-scale). From top to bottom, the rows increase the ratios of immunogenicity (${\omega}_2/{\omega}_1=0.1,2.2,10$), and the columns, from left to right, increase the ratios of immune-mediated elimination rates (${a}_2/{a}_1=0.1,1,10$), with fixed ${\omega}_1=0.001$, ${a}_1=0.01$, and the viral production ratio (${k}_2/{k}_1=1$). The colour transition from blue to red indicates increasing $R\left({\varepsilon}_1,{\varepsilon}_2\right)$ values. Panels (a)–(c) show an increase in $R\left({\varepsilon}_1,{\varepsilon}_2\right)$ towards the upper-right corner at ${\omega}_2/{\omega}_1=0.1$. Panels (g)–(i) show an opposite trend with $R\left({\varepsilon}_1,{\varepsilon}_2\right)$ increasing towards the lower-left corner at ${\omega}_2/{\omega}_1=10$. Panels (d)–(f) show a transitional pattern at ${\omega}_2/{\omega}_1=2.2$. Note that (e) displays a distinctive dual-peak pattern in $R\left({\varepsilon}_1,{\varepsilon}_2\right)$, located in the upper and lower-left regions.

Building upon our previous analyses, we focused on the fitness landscapes of ${V}_{total}$ [Equation (2)] and $R\left({\varepsilon}_1,{\varepsilon}_2\right)$ [Equation (5)] to assess the impact of different immune responses, specifically immunogenicity (${\omega}_2/{\omega}_1$) and immune-mediated elimination rates (${a}_2/{a}_1$), on optimizing oncogenic effects such as the transformation rate (${\varepsilon}_1$) and the proliferation rate (${\varepsilon}_2$). When pre-cancerous cells had low immunogenicity (${\omega}_2/{\omega}_1=0.1$), higher transformation and proliferation rates led to an increase in ${V}_{total}$ and $R\left({\varepsilon}_1,{\varepsilon}_2\right)$ (upper-right regions in Figs 5a–c and 6a–c), because a larger pre-cancerous cell population produced more virions and contributed more to total viral production [see Supplementary Information Section (f) and Supplementary Fig. S9a–c]. In contrast to these results obtained with a linear function for host infectiousness [$B(t)$] at the between-host scale, using a saturation function for $B(t)$ did not increase $R\left({\varepsilon}_1,{\varepsilon}_2\right)$ under higher transformation and proliferation rates since $B(t)$ reaches its maximum value in this higher oncogenic region, resulting in an upper bound on viral transmission (see Supplementary Figs S7-1a–c and S7-2a–c). On the other hand, if pre-cancerous cells were assumed to have high immunogenicity (${\omega}_2/{\omega}_1=10$), higher ${V}_{total}$ and $R\left({\varepsilon}_1,{\varepsilon}_2\right)$ were observed in regions of low oncogenicity, characterized by lower transformation and proliferation rates (lower-left regions in Figs 5g–i and 6g–i), as infected cells [$I(t)$] became the main source of viral production (Supplementary Fig. S9g–i). In these immunogenicity ranges, changing the immune-mediated elimination rates (${a}_2/{a}_1$) did not significantly impact the shape of the fitness landscape, indicating that immunogenicity affects viral dynamics more than immune-mediated elimination rates. Additionally, similar results were observed in a low-virulence scenario ($m=0.1$; see Supplementary Fig. S4-1), whereas in a high-virulence scenario ($m=5$), the trade-off between transmission and virulence changed the shape of the fitness landscape of $R\left({\varepsilon}_1,{\varepsilon}_2\right)$, shifting the optimal proliferation rate from low to intermediate levels around ${\varepsilon}_2=1$ (see Fig. S4-2i).

Notably, in cases of intermediate immunogenicity (${\omega}_2/{\omega}_1=1.23$ in Fig. 5d–f and ${\omega}_2/{\omega}_1=2.2$ in Fig. 6d–f), the fitness landscape showed a transition in the region of maximum ${V}_{total}$ and $R\left({\varepsilon}_1,{\varepsilon}_2\right)$ from the upper-right (= high oncogenicity region) to the lower-left (= low oncogenicity region). As shown in Figs 5e and 6e for ${a}_2/{a}_1=1$, ${V}_{total}$ and $R\left({\varepsilon}_1,{\varepsilon}_2\right)$ became higher in regions with both high and low proliferation rates, corresponding to a fitness minimum as confirmed in Fig. 4b and e. The shapes of these fitness landscapes for ${V}_{total}$ and $R\left({\varepsilon}_1,{\varepsilon}_2\right)$ were also observed in the results for the HPV-specific model (Supplementary Figs S8-1 and S8-2, respectively). Additionally, when immunogenicity was equal in both cell types (${\omega}_2/{\omega}_1=1.0$), the fitness landscape indicated a transition in the region of maximum ${V}_{total}$ and $R\left({\varepsilon}_1,{\varepsilon}_2\right)$ from the high oncogenicity region to the low transformation region (see Supplementary Fig. S1). Although our analysis used a viral production ratio of ${k}_2/{k}_1=1$, varying this ratio resulted in different outcomes: for instance, high oncogenicity became advantageous across all contour maps at ${k}_2/{k}_1=10$ (see Supplementary Figs S2-1 and S2-2). We also explored the scenario where pre-cancerous cells produce fewer viral particles than infected cells (${k}_2/{k}_1=0.1$) and evade the immune system (${\omega}_2/{\omega}_1=0.1$). Under this scenario higher proliferation rates led to higher within-host total viral load and between-host reproduction number across a range of transformation rates (see Supplementary Figs S3-1a–c and S3-2a–c). In scenarios where pre-cancerous cells do not produce virions and can revert to virion-producing infected cells (as seen in the life cycle of specific oncogenic viruses such as KSHV and EBV), we found that under low reversion rates, both the within-host total viral load and the between-host reproduction number increase with decreasing transformation rate and/or proliferation rate. Under high reversion rates and immune evasion, maximum virus fitness within and between hosts are expected under high proliferation rates and intermediate transformation rates [see Supplementary Information Section (c) and Supplementary Figs S6-1, S6-2, and S6-3]. Overall, these results provide a detailed analysis of the conditions under which oncogenicity enhances virus fitness within and between hosts.

## Discussion

Here, we have investigated the evolutionary conditions influencing viral oncogenesis. We have explored this through mathematical modelling and have focused on the oncovirus–immune system interaction. Our analysis explored the effects of viral oncogenicity, specifically the transformation rate of infected cells into pre-cancerous states and proliferation rate of pre-cancerous cells, on within-host viral dynamics (Fig. 2). Our results reveal an optimal transformation rate that maximizes both the within-host total viral load and the between-host reproduction number (measures of within- and between-host virus fitness, respectively), depending on three key factors: the viral production rate (Fig. 3a and d), immunogenicity (Fig. 3b and e), and the immune-mediated elimination rates of pre-cancerous cells (Fig. 3c and f). Under certain conditions, we also discovered intermediate proliferation rates of pre-cancerous cells that negatively affect virus fitness, which also vary with the viral production rate (Fig. 4a and d) and immunogenicity of pre-cancerous cells (Fig. 4b and e). We note that our results indicated that the immunogenicity of pre-cancerous cells plays a crucial role in determining the optimal viral oncogenicity (Figs 5 and 6). Through this ecological modelling approach, our analysis provides insights into the evolutionary conditions driving viral oncogenicity by describing an apparent competitive dynamic between infected and pre-cancerous cells mediated by the immune system and virus dynamics.

Oncogenicity is a double-edged sword for viruses. While the proliferation of infected cells may increase viral production, it also raises the risk of early detection and elimination by the immune system. Furthermore, when infected cells become fully cancerous, it results in the host’s death, ending the opportunity for viruses to transmit to new hosts. This can be considered an ‘evolutionary dead-end’ (Alizon et al. 2019, Murall and Alizon 2019) or ‘biological accident’ (Moore and Chang 2010, Chang et al. 2017) for viruses. Consequently, the degree of oncogenicity is under selective pressure leading to an optimal balance based on the environment. Our analyses showed that under unfavourable conditions for pre-cancerous cells, specifically when (i) their viral production rate is slightly low, (ii) immunogenicity is marginally high, and (iii) immune-mediated elimination is moderately high, an optimal transformation rate into pre-cancerous states can exist for maximizing both the within-host total viral load and the between-host reproduction number. Although there can be a small difference in the within-host viral load at these optimized parameter values compared to other parameter combinations, this finding is qualitatively consistent with previous studies (Murall et al. 2015), suggesting that oncogenic viruses may evolve traits that balance the advantages and disadvantages of oncogenicity.

In contrast to the fitness maximum found for the transformation rates into pre-cancerous cells, we also identified a fitness minimum for the proliferation rates of pre-cancerous cells, indicating that both low and high proliferation rates, rather than moderate ones, can be advantageous for maximizing both the within-host total viral load and the between-host reproduction number. This suggests one mechanism underlying the observed diversity of oncogenic viruses as fitness minima are likely to drive disruptive selection (Lord and Bonsall 2021). Lower proliferation rates can delay the immune responses, thereby increasing the within-host total viral load. Conversely, higher proliferation rates may lead to faster immune detection but strive to maximize the within-host total viral load before complete eradication by the immune system. As noted above, these contrasting strategies may contribute to the diversity observed in oncogenic viruses. For example, there is a wide range of HPV types from low to high risk that cause cervical cancer, which may result from differences in oncoprotein properties affecting variations in proliferation strategies (Schiffman et al. 2016). In a different approach to previous studies that linked transformation and proliferation rates of pre-cancerous cells in their model (Murall et al. 2015), our current work evaluates these oncogenic effects as separate parameters, revealing a fitness minimum in proliferation rates that provides a potential insight into the diversity of virulence among oncogenic viruses.

By comparing the shapes of the fitness landscape of ${V}_{total}$ and $R\left({\varepsilon}_1,{\varepsilon}_2\right)$, our results suggest that optimal viral oncogenicity within a host also maximizes the between-host virus fitness under two assumptions. First, the similarity in the shapes of the fitness landscape between different scales may arise from our model choice that the host infectiousness function is linearly proportional to the within-host viral load. Previous studies (Gilchrist and Coombs 2006) have demonstrated different scenarios in which the optimal strategies for within- and between-host virus fitness may accord or conflict, depending on the structure of the transmission and virulence terms, and our results are consistent with one of these scenarios. Although the relationship between viral load and host infectiousness remains unclear for oncogenic viruses, previous research on HIV-1 (Fraser et al. 2007) and SARS-CoV-2 (Ke et al. 2021) suggests a nonlinear relationship, and future studies should assess the consequences of this relationship to oncogenic viruses. Second, the influence of the virulence term [Equation (4)] directly affects between-host virus fitness in our model. In cases of lower virulence (e.g. $m=0.1$, see Supplementary Fig. S4-1), the within-host total viral load reflects more the viral transmission at a between-host scale, suggesting weaker selection pressure at the between-host level compared to the within-host scale. There are many approaches linking within- and between-host dynamics (Mideo et al. 2008, Doran et al. 2023), each requiring careful consideration. While we used the nested model commonly used in studies of virulence evolution, future research could aim to relax these assumptions to adjust the transmission and virulence term, and focus more on the biological characteristics of specific oncogenic viruses.

Our analysis also compared different models tailored to the different life cycles of representative oncogenic viruses, such as the small DNA viruses (e.g. oncogenic HPVs) and the large DNA viruses (e.g. KSHV and EBV). Although we used different parameter ranges for ${\varepsilon}_1$ and ${\varepsilon}_2$ in the fitness landscapes of ${V}_{total}$ and $R\left({\varepsilon}_1,{\varepsilon}_2\right)$, the shapes of these fitness landscapes of ${V}_{total}$ and $R\left({\varepsilon}_1,{\varepsilon}_2\right)$ remained similar in both the general model (Figs 5 and 6) and the HPV-specific model (Supplementary Figs S8-1 and S8-2). This similarity suggests that viral oncogenicity is more promoted when the immunogenicity of pre-cancerous cells is lower, consistent with the occurrence of high risk of virus-associated cancers in immunocompromised hosts [e.g. people living with HIV/AIDS and solid organ transplant recipients (Jin et al. 2024)]. Interestingly, this viral oncogenicity pattern also holds even if pre-cancerous cells do not produce virions, as observed from the results using the large DNA virus-specific model under a high reversion rate from non-productive to virion-producing states (i.e. ${\omega}_2<{\omega}_1$ and ${\varepsilon}_3=10$; Supplementary Fig. S6). This further suggests a theoretical explanation for why the large DNA viruses switch between lytic and latent phases in the context of viral evolution. Our approach may enhance our understanding of the diverse life cycles between different oncogenic viruses by focusing on the evolution of viral oncogenicity.

Our simulations further demonstrated that the immunogenicity of pre-cancerous cells has a significant impact on the fitness landscape of the within-host total viral load and the between-host reproduction number. Within the framework of the apparent competition among oncovirus–immune interactions, the immunogenicity of each virion-producing cell is a crucial step in triggering the immune system. This activated immune response eliminates each virion-producing cell, indicating that the within-host total viral load is regulated by a negative feedback mechanism rooted in the immunogenicity of individual virion-producing cells. Consequently, high immunogenicity of pre-cancerous cells negatively influences the within-host total viral load, while low immunogenicity tends to promote viral oncogenicity. Previous research into apparent competition between two parasite species and a shared immune system has shown how different functions of the immune response curve influence the community stability of two parasite environments (Fenton and Perkins 2010). While our model differs from this study (Fenton and Perkins 2010) in terms of the interaction structures between different types of infected cells and the immune system, our results extend the application of the apparent competition framework by highlighting the importance of interactions between the pathogens and the immune system.

Since mathematical modelling always requires simplifying assumptions to be made, our analysis has inherent limitations. First, our model does not account for the stochasticity of viral reactivation from latent infected cells and cancer initiation by oncogenic viruses, which is influenced by cellular proliferation. In the HPV-specific model, e.g. we assumed that oncogenic HPV infections are often asymptomatic and avirulent before cervical cancer develops (i.e. $m=0$), thus not accounting for the effects of cancer initiation and potentially underestimating the additional mortality of oncogenic HPVs at the between-host level. In addition, stochastic viral reactivation from latency is an important process of the oncogenicity for large DNA oncogenic viruses when host immunity declines. Since cancer initiation is also driven by chronic immune inflammation and increased mutation rates, further research is needed to address these factors and incorporate stochasticity as emphasized in previous studies (Murall and Alizon 2019).

Second, our model assumes uniform death rates for target cells, infected cells and pre-cancerous cells without considering the cytopathic effects of viruses and cell immortalization by the oncogenic viruses. In addition, our model does not explicitly represent the cell proliferation of infected cells [$I(t)$]. We expect that changing the death rates of pre-cancerous cells due to oncogenic effects would not alter our qualitative conclusions, as this effect is functionally equivalent to changing the proliferation rates of pre-cancerous cells in our mathematical model. However, considering the potential influence of both the cytopathic effects of viruses and cell immortalization on viral latency and reactivation in persistent infections, further research is needed to enhance our understanding of how these factors affect virus fitness.

Third, we defined the viral production rate of pre-cancerous cells (${k}_2$) as the product of the fixed cell death rate and the viral burst size, and assumed that ${k}_2$ was constant over time. In oncogenic HPVs, however, the viral production from pre-cancerous cells declines and shifts towards abortive infection as infected cells progress to a cancerous status (Schiffman et al. 2016), implying a decrease in the contribution of pre-cancerous cells to total viral production. To capture this transition to abortive infections, future refinements to our model should introduce an immortalized cell class that does not produce virions or allow the viral production rate of pre-cancerous cells (${k}_2$) to decrease over time.

Fourth, our model focuses on cell-mediated immunity, assuming complete elimination of viral infections, and does not consider a carrying capacity for immune cells and the effects of detailed immune evasion (e.g. immune suppression and T-cell exhaustion). Viral oncoproteins can suppress immune responses in various ways, such as altering cytokine/chemokine signalling, inhibiting HLA expression or repressing T-cell recruitment pathways (Roetman et al. 2022). Furthermore, immune effector cells often become dysfunctional by overexpression of inhibitory receptors such as PD-1 under chronic antigenic stimulation [e.g. chronic viral infections and tumour microenvironment (Jiang et al. 2015)]. These immune evasion strategies by oncogenic viruses allow infected cells to persist, resulting in the failure of viral and cancer clearance. In our model, we simplified the interplay among oncogenic effects (${\varepsilon}_1$ and ${\varepsilon}_2$), immunogenicity (${\omega}_2$), and the immune-mediated elimination rate (${a}_2$) for pre-cancerous cells by treating these parameters as independent. An important extension of our model would be to incorporate, more explicitly, immune evasion, such as considering immunogenicity (${\omega}_2$) or the immune-mediated elimination rate (${a}_2$) as a function of an oncogene expression level, so that ${\omega}_2$ (or ${a}_2$) decreases linearly or nonlinearly as the oncogene expression level increases. Under such scenarios, viral oncogenicity would be favoured because pre-cancerous cells with a higher proliferation rate could effectively evade immune surveillance and contribute more to viral production. However, a higher level of oncogene expression may drive infected cells to a cancerous state, thereby increasing host mortality. Taken together, these refinements enable a more detailed investigation of the trade-off between the advantages and disadvantages of oncogene expression at both within- and between-host scales.

Lastly, our model describes within-host viral dynamics and does not consider the details of between-host transmission dynamics, such as the heterogeneity of host populations, spatial structure, co- and super-infection, and host co-evolution. To address these limitations, employing multi-scale models that are more specific to the life cycles of each oncogenic virus could be beneficial for investigating the effects of persistent infection and immune suppression on virus fitness. Previous studies have utilized multi-scale modelling to explore the epidemiological and evolutionary dynamics of viral infections (Lythgoe et al. 2013, Hart et al. 2020, Schreiber et al. 2021, Doran et al. 2023, Sunagawa et al. 2023), and further research could incorporate these frameworks to evaluate the evolutionary consequences of viral oncogenicity.

In summary, the quantitative framework developed in this study is a step towards furthering our understanding of the evolutionary dynamics and drivers of oncogenic viruses within hosts. As we showed, this framework can be used to explore the impact of oncogenicity on virus fitness. There have been substantial recent advances in quantitative research in the fields of viral infections (Zitzmann and Kaderali 2018, Perelson and Ke 2021) and cancer (Altrock et al. 2015, Bozic and Wu 2020, McDonald et al. 2023), aiming at understanding biological phenomena as well as improving clinical practice. Mathematical modelling has the potential to generate further insights into the dynamics of oncogenic viruses, potentially elucidating evolutionary principles behind viral oncogenicity and leading to clinical improvement through further research.

## Supplementary Material

SI_veaf043

## Data Availability

Simulation codes for the data in this article are available at OSF: https://osf.io/erp97/?view_only=bc087c5bdca64d3d879f9c64864f5321/.
