# Supplementary material for: Within- and between-host evolutionary effects on viral oncogenicity
Source: Virus Evol. 2025 Jun 6;11(1):veaf043. doi: 10.1093/ve/veaf043 (PMC12218219; doi:10.1093/ve/veaf043)
Supplement: SI_veaf043 [file si_veaf043.docx]

**Supplementary Information to**

**“Within- and between-host evolutionary effects on viral oncogenicity”**

**Supplementary Results**

1. **Effects of different viral production rates of pre-cancerous cells on oncogenic outcomes**

To investigate effects of pre-cancerous cells on oncogenic advantages, we examined the effects of variation in viral production ratio (${k_{2}}/{k_{1}}$) on both within-host total viral load ($V_{total}$) and the between-host reproduction number [$R\left( \varepsilon_{1}, \varepsilon_{2} \right)$]. Since the cell death rate ($\delta$) is fixed in our analysis, adjusting $k_{i}$ corresponds to adjusting the viral burst size. Figures S2 and S3 illustrate scenarios of high (${k_{2}}/{k_{1}}=10$) and low (${k_{2}}/{k_{1}}=0.1$) viral production ratios, respectively. Figures S2-1&2 show that high oncogenic effects, characterized by high transformation and proliferation rates of pre-cancerous cells, correlate with both higher within-host total viral load and higher between-host reproduction number, regardless of immunogenicity or immune-mediated elimination rates of pre-cancerous cells. This suggests that the benefits of producing more virions from pre-cancerous cells outweigh the disadvantages of early elimination by the immune system.

In contrast, Figures S3 show an opposite trend with a low viral production ratio (${k_{2}}/{k_{1}}=0.1$). When pre-cancerous cells have high immunogenicity (Figs. S3-1&2d to S3-1&2i), low oncogenic effects, defined by lower transformation and proliferation rates, lead to higher within-host total viral load and between-host reproduction number. In this case, oncogenic traits become less favourable due to the increased risk of initiating an immune response. In addition, we observed the transition in Figures S3-1&2a to S3-1&2d, where the optimal region for increasing the within-host total viral load and the between-host reproduction number moves from low oncogenicity areas (lower-left in Figs. S3-1&2d) to regions of higher proliferation rates (upper in Figs. S3-1&2a). This transition highlights a strategic shift in the fitness landscape: when lower viral production from pre-cancerous cells coincides with low immunogenicity, it becomes more advantageous to maintain viral production from infected cells at a lower cell transformation rate while increasing the proliferation rate of pre-cancerous cells.

1. **Sensitivity to the additional mortality rates due to viral infections**

As the value of the additional mortality rates due to viral infection ($m$) is unknown, we assumed $m=1.0$ as the baseline for virulence and conducted simulations with different values of $m$: $m=0.1$ for a low-virulence case (Fig. S4-1) and $m=5$ for a high-virulence case (Fig. S4-2). In Fig. S4-1, the shapes of the fitness landscape of $R\left( \varepsilon_{1}, \varepsilon_{2} \right)$ are similar to that of $V_{total}$ (Fig. 5 in the main text). This is because the viral virulence is low and has little effect on reducing viral transmission at the between-host level. As a result, the optimal oncogenic effects that maximise the within-host virus fitness also maximise the between-host fitness. In contrast, when the viral virulence is high ($m=5$), the shapes of the fitness landscape of $R\left( \varepsilon_{1}, \varepsilon_{2} \right)$ become different from that of $V_{total}$. In Fig. S4-1i, for instance, $R\left( \varepsilon_{1}, \varepsilon_{2} \right)$ is maximised in the lower-left corner (= low oncogenic area), whereas in Fig. S4-2i, $R\left( \varepsilon_{1}, \varepsilon_{2} \right)$ decreases in the same region and is instead maximised around $\varepsilon_{2}=1$, suggesting that the trade-off between transmission and virulence is balanced in this area. These results indicate that as the degree of viral virulence ($m$) changes, the optimal oncogenic effects that maximise virus fitness at the within- and between-host levels become different. Thus, in our model, the degree of viral virulence determines whether the optimal oncogenic effects that maximise virus fitness converge or diverge across different scales.

1. **The different life cycles of oncogenic viruses such as KSHV and EBV**

We modified the original model of oncogenic viruses in Equation (1) to capture the different life cycle of certain oncogenic viruses such as KSHV and EBV. This modification addressed the scenarios where pre-cancerous cells do not produce virions and return to virion-producing infected cells as follows:

(S1)

$$\frac{dT\left( t \right)}{dt}=\lambda-\delta T\left( t \right)-\beta V\left( t \right)T\left( t \right),$$

$$\frac{dI\left( t \right)}{dt}=\beta V\left( t \right)T\left( t \right){+\varepsilon}_{3}P\left( t \right)-\varepsilon_{1}I\left( t \right)-a_{1}I\left( t \right)E\left( t \right)-\delta I\left( t \right),$$

$$\frac{dP\left( t \right)}{dt}=\varepsilon_{1}I\left( t \right)+\varepsilon_{2}P\left( t \right)-a_{2}P\left( t \right)E\left( t \right)-\varepsilon_{3}P\left( t \right)-\delta P\left( t \right),$$

$$\frac{dV\left( t \right)}{dt}=k_{1}I\left( t \right)-cV\left( t \right),$$

$$\frac{dE(t)}{dt}=\omega_{1}I\left( t \right)E\left( t \right)+\omega_{2}P\left( t \right)E\left( t \right).$$

In this model, we assumed that no virion is produced by the pre-cancerous cells, i.e. $k_{2}=0$ in Equation (1), and pre-cancerous cells revert to virion-producing infected cells at the reversion rate $\varepsilon_{3}$ (Fig. S5).

Using Equations (S1) and (5), we calculated the fitness landscapes of $V_{total}$​ and $R\left( \varepsilon_{1}, \varepsilon_{2} \right)$ to assess the impact of immunogenicity (${\omega_{2}}/{\omega_{1}}$) and reversion rates ($\varepsilon_{3}$​) on optimising oncogenic effects such as the transformation rate ($\varepsilon_{1}$​) and the proliferation rate ($\varepsilon_{2}$). When pre-cancerous cells did not revert to virion-producing infected cells ($\varepsilon_{3}=0$) or had low reversion rates ($\varepsilon_{3}=1.0$), lower transformation and proliferation rates led to an increase in $V_{total}$​ (lower-left regions in Figs. S6-1a&b). This is because pre-cancerous cells do not contribute to viral production and instead trigger the immune response, leading to the indirect elimination of virion-producing infected cells. Conversely, when pre-cancerous cells had high reversion rates ($\varepsilon_{3}=10$) and low immunogenicity (${\omega_{2}}/{\omega_{1}}=0.1$), higher $V_{total}$ was observed in regions with higher proliferation rates and intermediate transformation rates (central upper regions in Fig. S6-1c). This occurs because the proliferated pre-cancerous cells evaded the immune system, reverted to virion-producing infected cells, and contributed to viral production. If the immunogenicity of pre-cancerous cells is higher (${\omega_{2}}/{\omega_{1}}=1.23$), virion-producing infected cells are removed by the immune system induced by pre-cancerous cells, suggesting that lower proliferation and transformation rates optimise the within-host total viral load (lower-left regions in Figs. S6-1d to S6-1i). Therefore, the balance between the immunogenicity and the reversion rates of pre-cancerous cells determines the optimal transformation and proliferation rates for maximising the within-host total viral load.

As described in the sensitivity analysis of the additional mortality rates $m$ in section (b), varying $m$ values affect the optimal oncogenic effects that maximise the between-host virus fitness in the different life cycle of oncogenic viruses. For example, in a low-virulence case ($m=0.1$), the shape of the fitness landscape of $R\left( \varepsilon_{1}, \varepsilon_{2} \right)$ becomes similar to that of $V_{total}$ (Figs. S6-1 and S6-2). On the other hand, at the baseline value for the viral virulence ($m=1.0$), the fitness landscape of $R\left( \varepsilon_{1}, \varepsilon_{2} \right)$ differs from that of $V_{total}$ (Figs. S6-3b&f). This result is consistent with the shape of the fitness landscape obtained using Equation (1) (Fig. S4-2i), emphasizing that the trade-off between transmission and virulence determines the optimal oncogenic effects that maximise $R\left( \varepsilon_{1}, \varepsilon_{2} \right)$ even for oncogenic viruses with the different life cycle such as KSHV and EBV.

1. **Effects of a nonlinear relationship between viral load and host infectiousness on oncogenic outcomes**

To evaluate how a nonlinear relationship between viral load and host infectiousness affects the fitness landscape of the reproduction number, we considered a nonlinear function for host infectiousness. Following previous studies on HIV-1 ([Fraser et al. 2007](#_ENREF_1)) and SARS-CoV-2 ([Ke et al. 2021](#_ENREF_2)), we defined host infectiousness [$B\left( t \right)$] using a Hill function of the viral load:

$$B\left( t \right)=\beta_{BH\_max}\frac{{V\left( \varepsilon_{1}, \varepsilon_{2},t \right)}^{h}}{{V\left( \varepsilon_{1}, \varepsilon_{2},t \right)}^{h}+{\beta_{50}}^{h}}, (S2)$$

where $h$ is the steepness of the curve, $\beta_{BH\_max}$ is the maximum infectious rate, and $\beta_{50}$ is the viral load at which infectiousness reaches half its maximum. Substituting Equation (S2) for $B\left( t \right)$ into Equation (3) for $R_{0}$, we defined the between-host reproduction number $R\left( \varepsilon_{1}, \varepsilon_{2} \right):$

$$R\left( \varepsilon_{1}, \varepsilon_{2} \right)=\int_{0}^{t_{end}} b\frac{{V\left( \varepsilon_{1}, \varepsilon_{2},t \right)}^{h}}{{V\left( \varepsilon_{1}, \varepsilon_{2},t \right)}^{h}+{\beta_{50}}^{h}}\exp\left( -\mu t-\mu m\int_{0}^{t} \left( T\left( 0 \right)-T(\varepsilon_{1}, \varepsilon_{2},z) \right)dz \right)dt, (S3)$$

where $b= \beta_{BH\_max}S_{0}$​ serves as a scaling factor. For simulation, we fixed $\beta_{50}={10}^{4}$ and chose the value of $b$ so that the between-host reproduction number of the virus without oncogenic effects is 1 [i.e. $R\left( 0, 0 \right)=1$]. In the case of $h=1$ (Fig. S7-1), the high oncogenic region with a higher viral peak is no longer optimal (the upper-right region in Fig. S7-1a–c), in contrast to the results using a linear function for host infectiousness (Fig. 6a–c). This difference occurs because once the viral load exceeds $\beta_{50}$, host infectiousness [$B\left( t \right)$] reaches its maximum value, resulting in saturated viral transmission as well. In the case of $h=2$ (Fig. S7-2), there is an optimal region in both the transformation rate and the proliferation rate that maximises $R\left( \varepsilon_{1}, \varepsilon_{2} \right)$. In Figure S7-2e, for example, $R\left( \varepsilon_{1}, \varepsilon_{2} \right)$ reaches its peak around the middle-left area (= intermediate proliferation rates). As the nonlinear relationship between viral load and infectiousness becomes more sigmoidal, higher viral load quickly reach a saturated viral transmission, while lower viral load results in reduced viral transmission. These results suggest that as the nonlinearity between viral load and host infectiousness increases, viral transmission saturates in higher oncogenicity regions where viral peak becomes high, and then the maximum between-host viral fitness shifts toward intermediate oncogenicity regions.

1. **HPV-specific model**

To focus on the HPV life cycle, we adapted the previous HPV-specific model developed by ([Murall et al. 2015](#_ENREF_3)) to our analysis. The form of the HPV-specific model is given by

$$\frac{dI\left( t \right)}{dt}=\beta V\left( t \right)\frac{N-I\left( t \right)}{\phi+\left( N-I\left( t \right) \right)}-\varepsilon I\left( t \right)-\delta I\left( t \right)-a_{1}I\left( t \right)E\left( t \right),$$

$$\frac{dP\left( t \right)}{dt}=\varepsilon I\left( t \right)+r\varepsilon P\left( t \right)-\delta P\left( t \right)-a_{2}P\left( t \right)E\left( t \right),$$

$$\frac{dV\left( t \right)}{dt}=\delta\left( k_{1}I\left( t \right)+k_{2}P\left( t \right) \right)-cV\left( t \right),$$

$$\frac{dE(t)}{dt}=\omega_{1}I\left( t \right)E\left( t \right)+\omega_{2}P\left( t \right)E\left( t \right),$$

where the variables $I(t)$, $P(t)$, $V(t)$ and $E(t)$ represent the populations of non-proliferating infected cells, proliferating infected cells (i.e. pre-cancerous cells), virus, and immune effector cells, respectively. $N$ is the total population of all epithelial cells, $\phi$ is the half-growth constant, $r$ is the self-division rate of infected cells, $\varepsilon$ is the rate of oncogene expression such as E6 and E7, and $k_{i}$ is the burst size of virions from infected cells. Infected cells die at the rate $\delta$, releasing virions at death, and the viral production rates depend on $\delta k_{i}$. The original model ([Murall et al. 2015](#_ENREF_3)) assumes a constant epithelial cell population $N$, and the target cell population [$T\left( t \right)$] is replaced by $N-I\left( t \right)$ in Equation (S4). HPV targets basal epithelial cells under the epithelium and requires abrasions for virion access, so the original model used a saturation function for HPV infections and defined $\phi$ as the uninfected cell density when the growth rate of $I\left( t \right)$ is its half-maximal [see ([Murall et al. 2015](#_ENREF_3)) for details].

(S4)

We adapted some of the original notation used for parameters and variables to match the terms used in our study. To distinguish the transformation rate from the proliferation rate, we denote the original transformation rate (oncogene expression rate $\varepsilon$) as $\varepsilon_{1}$ and define the self-proliferation of infected cells as $\varepsilon_{2}=r\varepsilon$. Since the original model set $r=0.1$, we varied $\varepsilon_{1}$ and $\varepsilon_{2}$ from 0.0001 to 1.0 and used the same parameter sets as in the original study for simulation [adapted from Table 1 in ([Murall et al. 2015](#_ENREF_3))]: infection rate ($\beta=0.0067$), death rate of cells ($\delta=0.048$), burst size ($k=1000$), decay rate of virions ($c=0.05$), proliferation rate of immune cells ($\omega=0.001$), killing rate of immune cells ($a=0.01$), total population of all epithelial cells ($N=10000$), half-growth constant ($\phi={10}^{6}$). Although the original model does not include the immunogenicity of non-proliferating infected cells, $\omega_{1}I\left( t \right)E\left( t \right)$, we incorporated this term to examine how apparent competition between non-proliferating and proliferating infected cells affects virus fitness at both within- and between-host scales.

Using the HPV-specific model, we calculated the fitness landscapes of $V_{total}$​ and $R\left( \varepsilon_{1}, \varepsilon_{2} \right)$ with different sets of immunogenicity (${\omega_{2}}/{\omega_{1}}$) and immune-mediated elimination rates (${a_{2}}/{a_{1}}$). For these simulations, we assumed no additional mortality from oncogenic HPVs [i.e. $m=0$ in Equation (5) for $R\left( \varepsilon_{1}, \varepsilon_{2} \right)$] since oncogenic HPV infections are often asymptomatic and avirulent [similar to ([Murall et al. 2015](#_ENREF_3))]. Interestingly, the shapes of fitness landscapes of $V_{total}$​ (Fig. S8-1) and $R\left( \varepsilon_{1}, \varepsilon_{2} \right)$ (Fig. S8-2) are similar to the results using the general model (Figs. 5 and 6, respectively). Although the absolute values of ​$V_{total}$​ and $R\left( \varepsilon_{1}, \varepsilon_{2} \right)$ differ from the results shown in Figures 5 and 6, the similarity in landscape shapes suggests that the qualitative conclusions hold under this HPV-specific model as well.

1. **The proportion of total viral production contributed by pre-cancerous cells**

We calculated the contribution of pre-cancerous cells to total viral production under different oncogenic effects ($\varepsilon_{1}$ and $\varepsilon_{2}$), immunogenicity ​(${\omega_{2}}/{\omega_{1}}$) and immune-mediated elimination rates (${a_{2}}/{a_{1}}$). We defined $V_{prod,P}$ as the total viral production by pre-cancerous cells during the infection, expressed as $V_{prod,P}=\int_{0}^{t_{end}} k_{2}P\left( t \right)dt$. To distinguish $V_{total}$ in the main text representing the area under the curve of $V\left( t \right)$ during the infection, we defined the total viral production from all infected cells as $V_{prod,total}=\int_{0}^{t_{end}} k_{1}I\left( t \right)+k_{2}P\left( t \right)dt$. We then defined the proportion of viral production attributable to pre-cancerous cells as $f_{p}={V_{prod,P}}/{V_{prod,total}}$.

Figure S9 shows that as oncogenic effects ($\varepsilon_{1}$ and $\varepsilon_{2}$) increase, $f_{p}$ also increases under different ratios of immunogenicity ​(${\omega_{2}}/{\omega_{1}}$) and immune-mediated elimination rates (${a_{2}}/{a_{1}}$). By comparing $R\left( \varepsilon_{1}, \varepsilon_{2} \right)$ in Figure 6e with $f_{p}$ in Figure S9e, we find that the primary source of viral production can differ depending on the region of the fitness landscape, even for the same $R\left( \varepsilon_{1}, \varepsilon_{2} \right)$. When $R\left( \varepsilon_{1}, \varepsilon_{2} \right)$ peaks in the upper-left region where pre-cancerous cells have a high proliferation rate, virions are predominantly produced by pre-cancerous cells ($f_{p}>0.8$). In contrast, when $R\left( \varepsilon_{1}, \varepsilon_{2} \right)$ peaks in the lower-left region (= low oncogenicity region), the contribution from pre-cancerous cells is minimal ($f_{p}<0.1$). Intuitively, a larger pre-cancerous cell population produces more virions and contributes more to total viral production. These results suggest that total viral production can arise from different infected cell populations, and its composition may vary depending on the growth rates of each virion-producing cell.


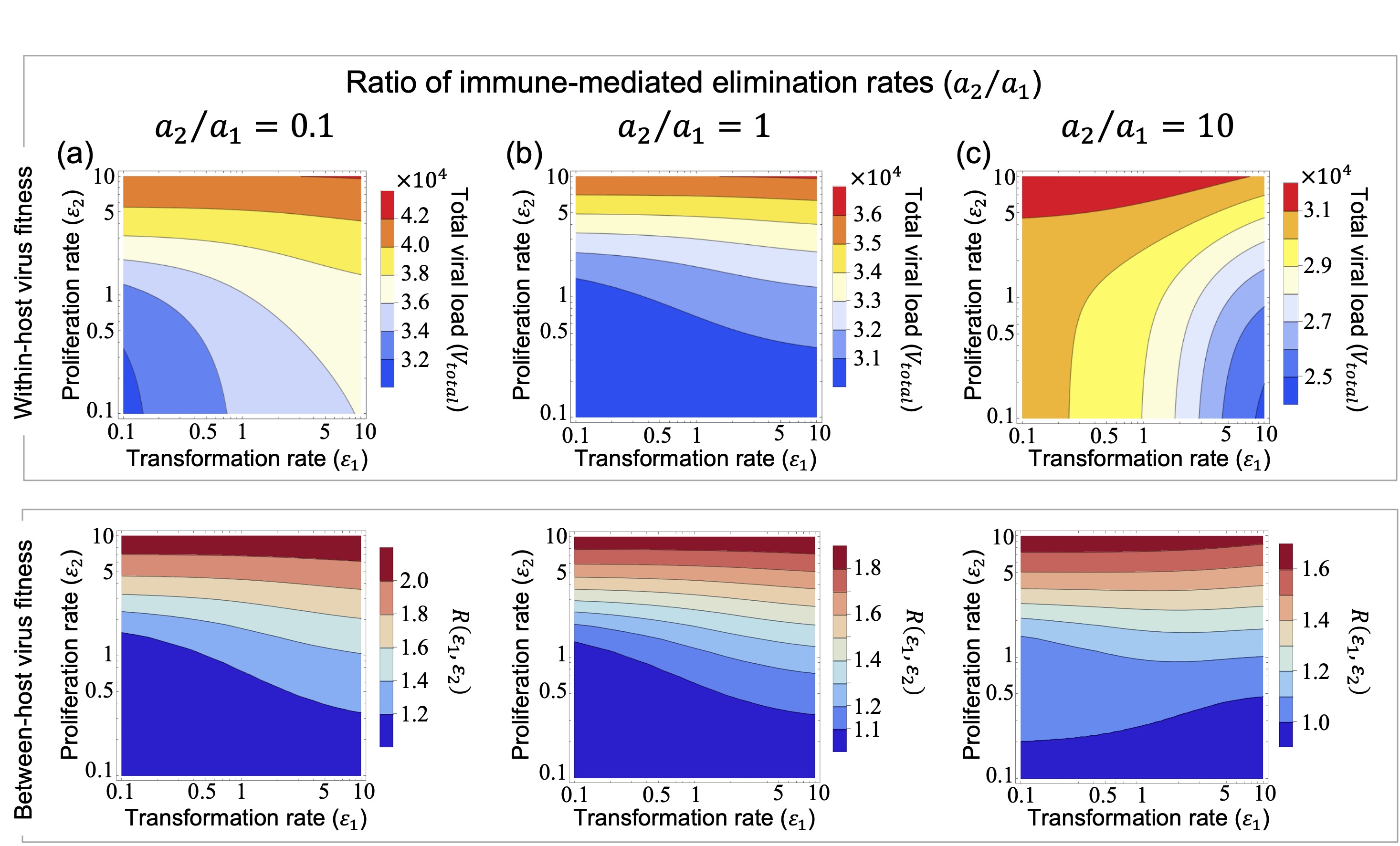
**Figure S1. Effects of varying transformation and proliferation rates on the within-host total viral load and the between-host reproduction number at different immune-mediated elimination rates of pre-cancerous cells.** Contour maps showing the changes in the within-host total viral load ($V_{total}$) and the between-host reproduction number [$R\left( \varepsilon_{1}, \varepsilon_{2} \right)$] in response to various ratios of immune-mediated elimination rates (${a_{2}}/{a_{1}}$) when infected cells and pre-cancerous cells have the same immunogenicity and viral production rates (${\omega_{2}}/{\omega_{1}}=1$ and ${k_{2}}/{k_{1}}=1)$, plotted against transformation rates ($\varepsilon_{1}$, x-axis, log-scale) and proliferation rates ($\varepsilon_{2}$, y-axis, log-scale). From left to right, the columns increase the ratios of immune-mediated elimination rates (${a_{2}}/{a_{1}}=0.1, 1, 10$) with fixed $a_{1}=0.01$. The colour transition from blue to red indicates increasing $V_{total}$ and $R\left( \varepsilon_{1}, \varepsilon_{2} \right)$ values. (a)-(c) show a transitional pattern where the region of higher $V_{total}$ and $R\left( \varepsilon_{1}, \varepsilon_{2} \right)$ shifts from the upper-right corner to the upper-left corner. In (a) and (b), when pre-cancerous cells have lower or equivalent immune-mediated elimination rates compared to infected cells, higher transformation and proliferation rates of pre-cancerous cells lead to a higher within-host total viral load. In contrast, (c) shows a situation when lower transformation rates are advantageous to avoid the high immune-mediated elimination of pre-cancerous cells, with increased cell proliferation compensating for the loss of pre-cancerous cells due to the immune response.

**
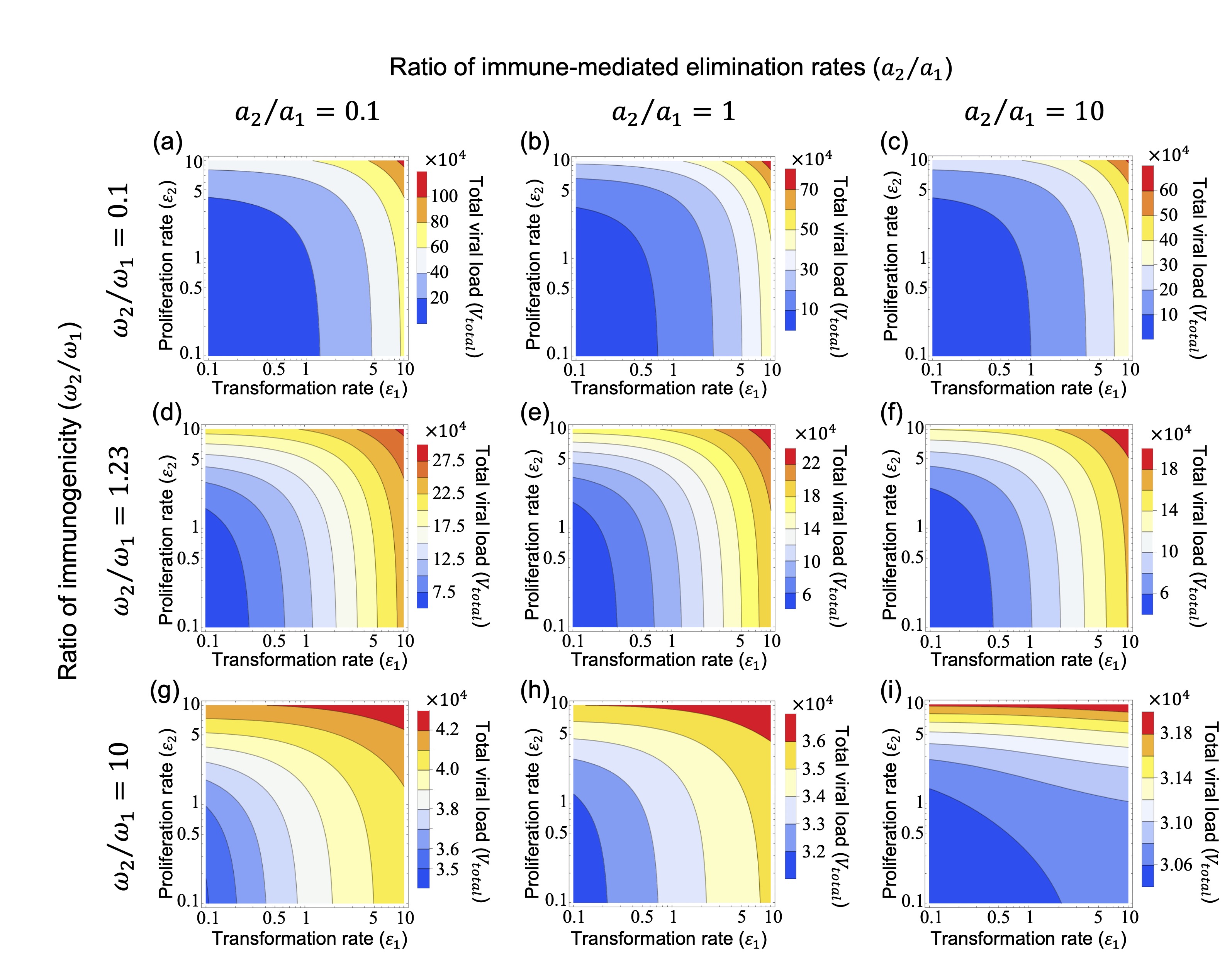
Figure S2-1. Effects of varying transformation and proliferation rates on the within-host total viral load at high viral production rates of pre-cancerous cells.** Contour maps showing the changes in the within-host total viral load ($V_{total}$) in response to various combinations of immunogenicity (${\omega_{2}}/{\omega_{1}}$) and immune-mediated elimination rates (${a_{2}}/{a_{1}}$) when viral production rates were higher in pre-cancerous cell $({k_{2}}/{k_{1}}=10)$, plotted against transformation rates ($\varepsilon_{1}$, x-axis, log-scale) and proliferation rates ($\varepsilon_{2}$, y-axis, log-scale). From top to bottom, the rows increase the ratios of immunogenicity (${\omega_{2}}/{\omega_{1}}=0.1, 1.23, 10$), and the columns, from left to right, increase the ratios of immune-mediated elimination rates (${a_{2}}/{a_{1}}=0.1, 1, 10$), with fixed $\omega_{1}=0.001$, $a_{1}=0.01$, and the viral production ratio (${k_{2}}/{k_{1}}=10$). The colour transition from blue to red indicates increasing $V_{total}$ values. (a)-(i) show an increase in $V_{total}$ towards the upper-right corner, regardless of the values of ${\omega_{2}}/{\omega_{1}}$ and ${a_{2}}/{a_{1}}$. **
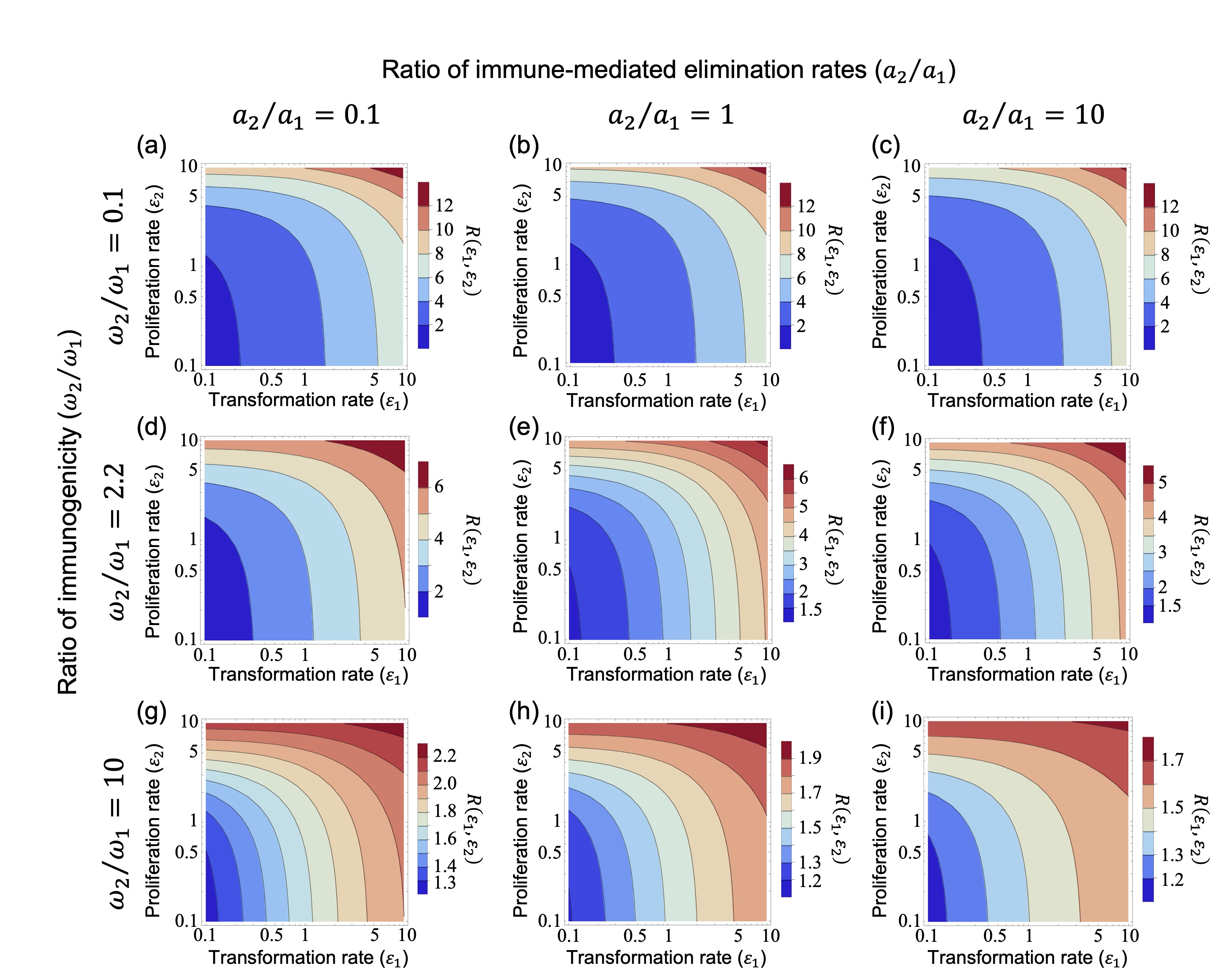
Figure S2-2. Effects of varying transformation and proliferation rates on the between-host reproduction number at high viral production rates of pre-cancerous cells.** Contour maps showing the changes in the between-host reproduction number [$R\left( \varepsilon_{1}, \varepsilon_{2} \right)$] in response to various combinations of immunogenicity (${\omega_{2}}/{\omega_{1}}$) and immune-mediated elimination rates (${a_{2}}/{a_{1}}$) when viral production rates were higher in pre-cancerous cells (${k_{2}}/{k_{1}}=10)$, plotted against transformation rates ($\varepsilon_{1}$, x-axis, log-scale) and proliferation rates ($\varepsilon_{2}$, y-axis, log-scale). From top to bottom, the rows increase the ratios of immunogenicity (${\omega_{2}}/{\omega_{1}}=0.1, 2.2, 10$), and the columns, from left to right, increase the ratios of immune-mediated elimination rates (${a_{2}}/{a_{1}}=0.1, 1, 10$), with fixed $\omega_{1}=0.001$, $a_{1}=0.01$, and the viral production ratio (${k_{2}}/{k_{1}}=10$). The colour transition from blue to red indicates increasing $R\left( \varepsilon_{1}, \varepsilon_{2} \right)$ values. (a)-(i) show an increase in $R\left( \varepsilon_{1}, \varepsilon_{2} \right)$ towards the upper-right corner, regardless of the values of ${\omega_{2}}/{\omega_{1}}$ and ${a_{2}}/{a_{1}}$.

**
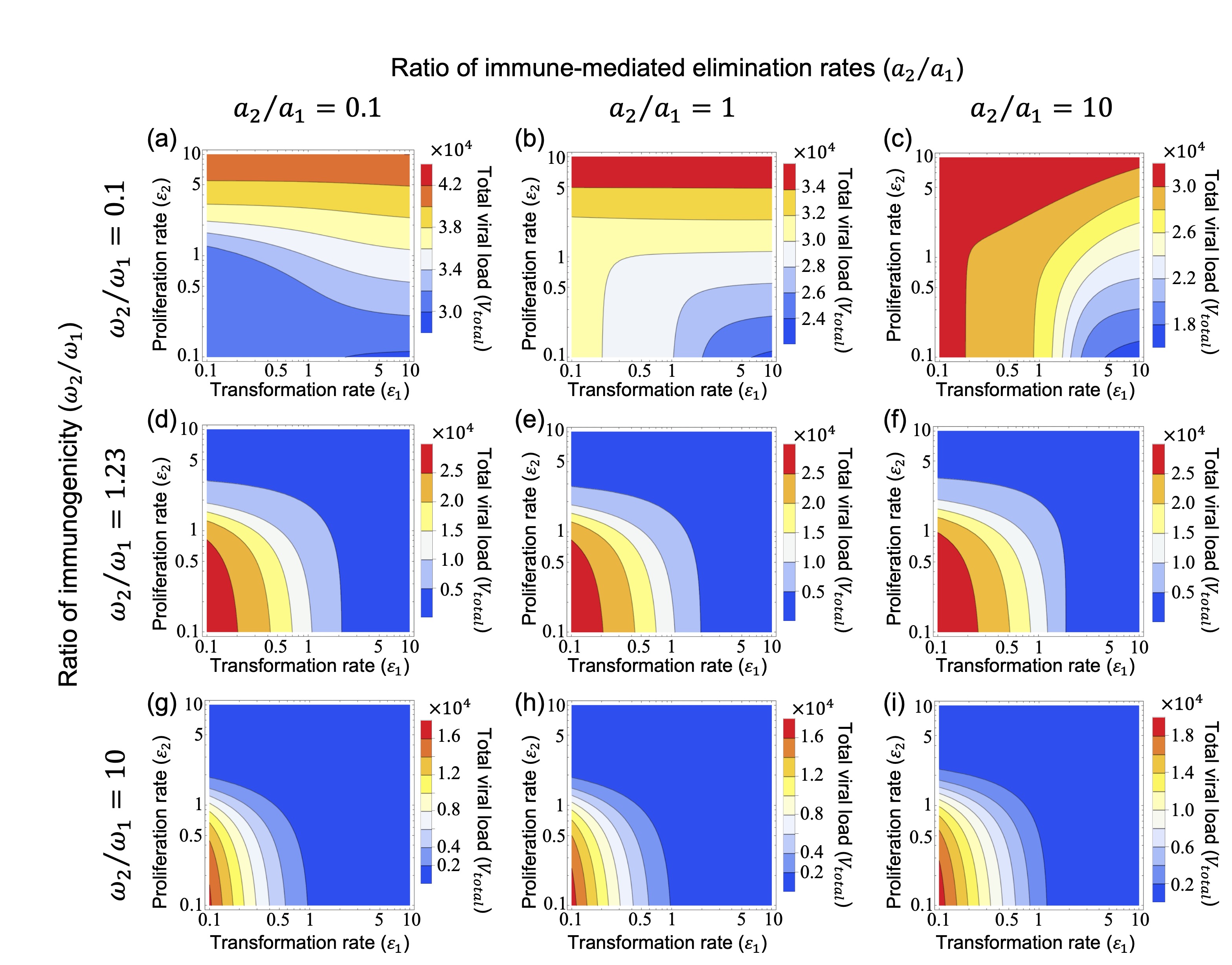
Figure S3-1. Effects of varying transformation and proliferation rates on the within-host total viral load at low viral production rates of pre-cancerous cells.** Contour maps showing the changes in the within-host total viral load ($V_{total}$) in response to various combinations of immunogenicity (${\omega_{2}}/{\omega_{1}}$) and immune-mediated elimination rates (${a_{2}}/{a_{1}}$) when viral production rates were lower in pre-cancerous cells (${k_{2}}/{k_{1}}=0.1)$, plotted against transformation rates ($\varepsilon_{1}$, x-axis, log-scale) and proliferation rates ($\varepsilon_{2}$, y-axis, log-scale). From top to bottom, the rows increase the ratios of immunogenicity (${\omega_{2}}/{\omega_{1}}=0.1, 1.23, 10$), and the columns, from left to right, increase the ratios of immune-mediated elimination rates (${a_{2}}/{a_{1}}=0.1, 1, 10$), with fixed $\omega_{1}=0.001$, $a_{1}=0.01$, and the viral production ratio (${k_{2}}/{k_{1}}=0.1$). The colour transition from blue to red indicates increasing $V_{total}$ values. (a)-(c) show a transitional pattern in the fitness landscape at ${\omega_{2}}/{\omega_{1}}=0.1$. (a) shows an increase in $V_{total}$ towards the upper-right corner. In (b), higher $V_{total}$ values are in the upper half area. (c) shows a trend with $V_{total}$ increasing towards the upper-left corner. (d)-(i) show an increase in $V_{total}$ towards the lower-left corner (= low oncogenic area) at ${\omega_{2}}/{\omega_{1}}=1.23$ and $10$.

**
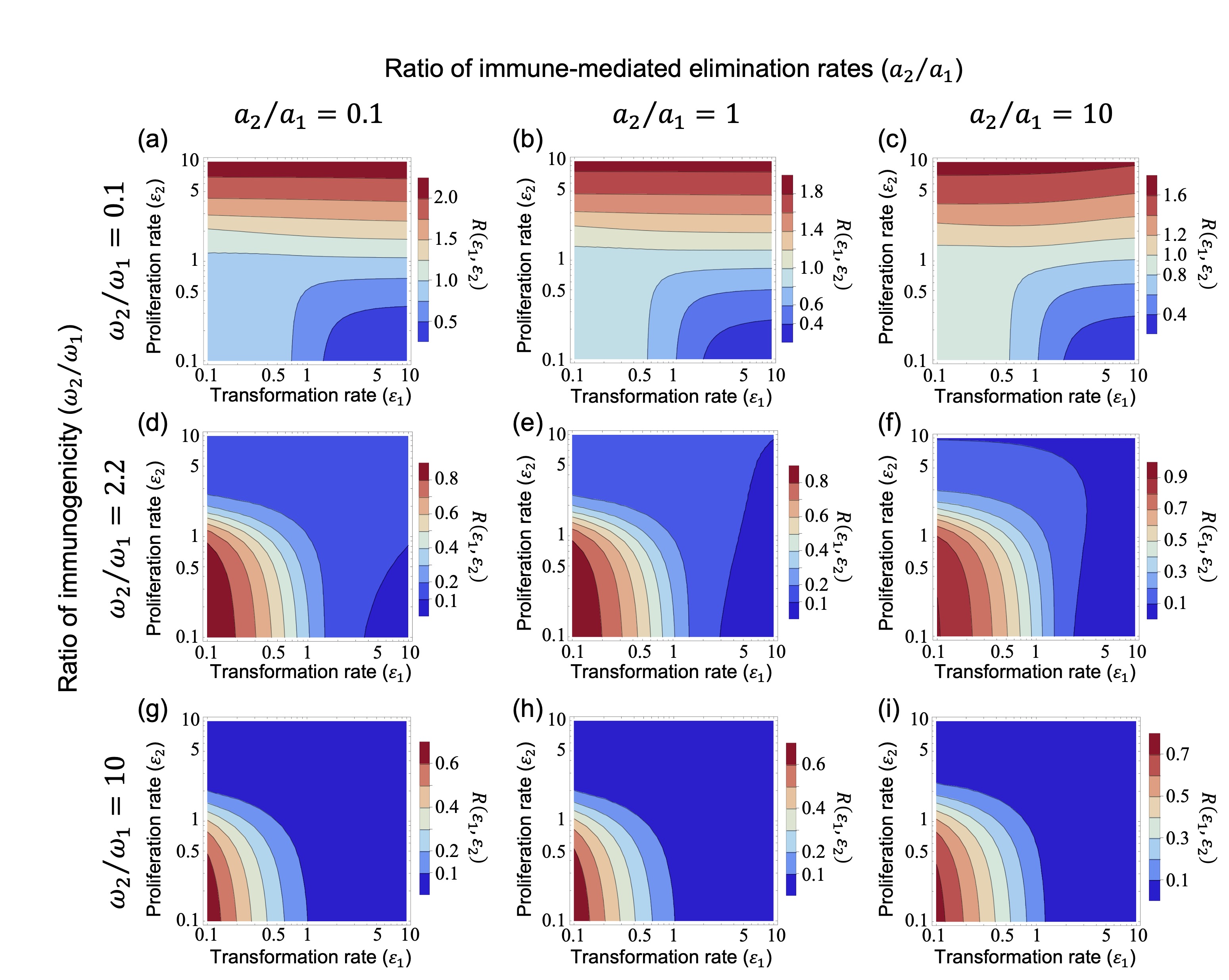
Figure S3-2. Effects of varying transformation and proliferation rates on the between-host reproduction number at low viral production rates of pre-cancerous cells.** Contour maps showing the changes in the between-host reproduction number [$R\left( \varepsilon_{1}, \varepsilon_{2} \right)$] in response to various combinations of immunogenicity (${\omega_{2}}/{\omega_{1}}$) and immune-mediated elimination rates (${a_{2}}/{a_{1}}$) when viral production rates were lower in pre-cancerous cells (${k_{2}}/{k_{1}}=0.1)$, plotted against transformation rates ($\varepsilon_{1}$, x-axis, log-scale) and proliferation rates ($\varepsilon_{2}$, y-axis, log-scale). From top to bottom, the rows increase the ratios of immunogenicity (${\omega_{2}}/{\omega_{1}}=0.1, 2.2, 10$), and the columns, from left to right, increase the ratios of immune-mediated elimination rates (${a_{2}}/{a_{1}}=0.1, 1, 10$), with fixed $\omega_{1}=0.001$, $a_{1}=0.01$, and the viral production ratio (${k_{2}}/{k_{1}}=0.1$). The colour transition from blue to red indicates increasing $R\left( \varepsilon_{1}, \varepsilon_{2} \right)$ values. (a)-(c) show an increase in $R\left( \varepsilon_{1}, \varepsilon_{2} \right)$ towards the upper half area of higher proliferation rates. (d)-(i) show a trend with increasing $R\left( \varepsilon_{1}, \varepsilon_{2} \right)$ towards the lower-left corner (= low oncogenic area) at ${\omega_{2}}/{\omega_{1}}=2.2$ and $10$.

**
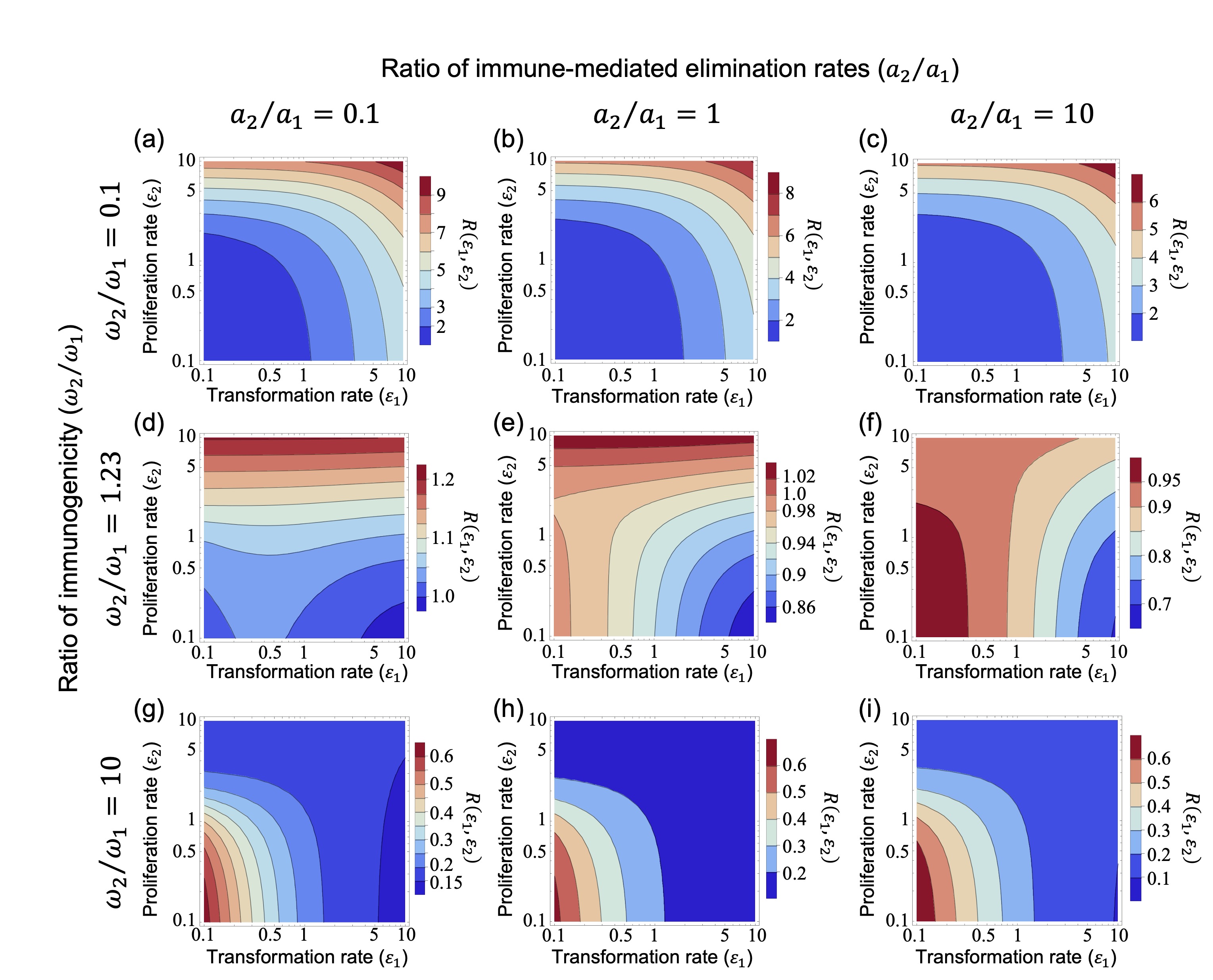
Figure S4-1. Effects of varying transformation and proliferation rates on the between-host reproduction number at the low additional mortality rate.** Contour maps showing the changes in the between-host reproduction number [$R\left( \varepsilon_{1}, \varepsilon_{2} \right)$] in response to various combinations of immunogenicity (${\omega_{2}}/{\omega_{1}}$) and immune-mediated elimination rates (${a_{2}}/{a_{1}}$) when the additional mortality rate was low ($m=0.1)$, plotted against transformation rates ($\varepsilon_{1}$, x-axis, log-scale) and proliferation rates ($\varepsilon_{2}$, y-axis, log-scale). From top to bottom, the rows increase the ratios of immunogenicity (${\omega_{2}}/{\omega_{1}}=0.1, 1.23, 10$), and the columns, from left to right, increase the ratios of immune-mediated elimination rates (${a_{2}}/{a_{1}}=0.1, 1, 10$), with fixed $\omega_{1}=0.001$, $a_{1}=0.01$, and the viral production ratio (${k_{2}}/{k_{1}}=0.1$). We set $b={3.5126\times10}^{-5}$ so that the between-host reproduction number of the virus without oncogenic effects is 1 [i.e. $R\left( 0,0 \right)=1$]. The colour transition from blue to red indicates increasing $R\left( \varepsilon_{1}, \varepsilon_{2} \right)$ values. (a)-(c) show an increase in $R\left( \varepsilon_{1}, \varepsilon_{2} \right)$ towards the upper-right corner (= high oncogenic area) at ${\omega_{2}}/{\omega_{1}}=0.1$; (g)-(i) show an opposite trend with $R\left( \varepsilon_{1}, \varepsilon_{2} \right)$ increasing towards the lower-left corner (= low oncogenic area) at ${\omega_{2}}/{\omega_{1}}=10$. (d)-(f) show a transitional pattern in the fitness landscape at ${\omega_{2}}/{\omega_{1}}=1.23$. Note that when the virulence of viral infections is low ($m=0.1)$, the shape of fitness landscape of $R\left( \varepsilon_{1}, \varepsilon_{2} \right)$ correlates with that of $V_{total}$.

**
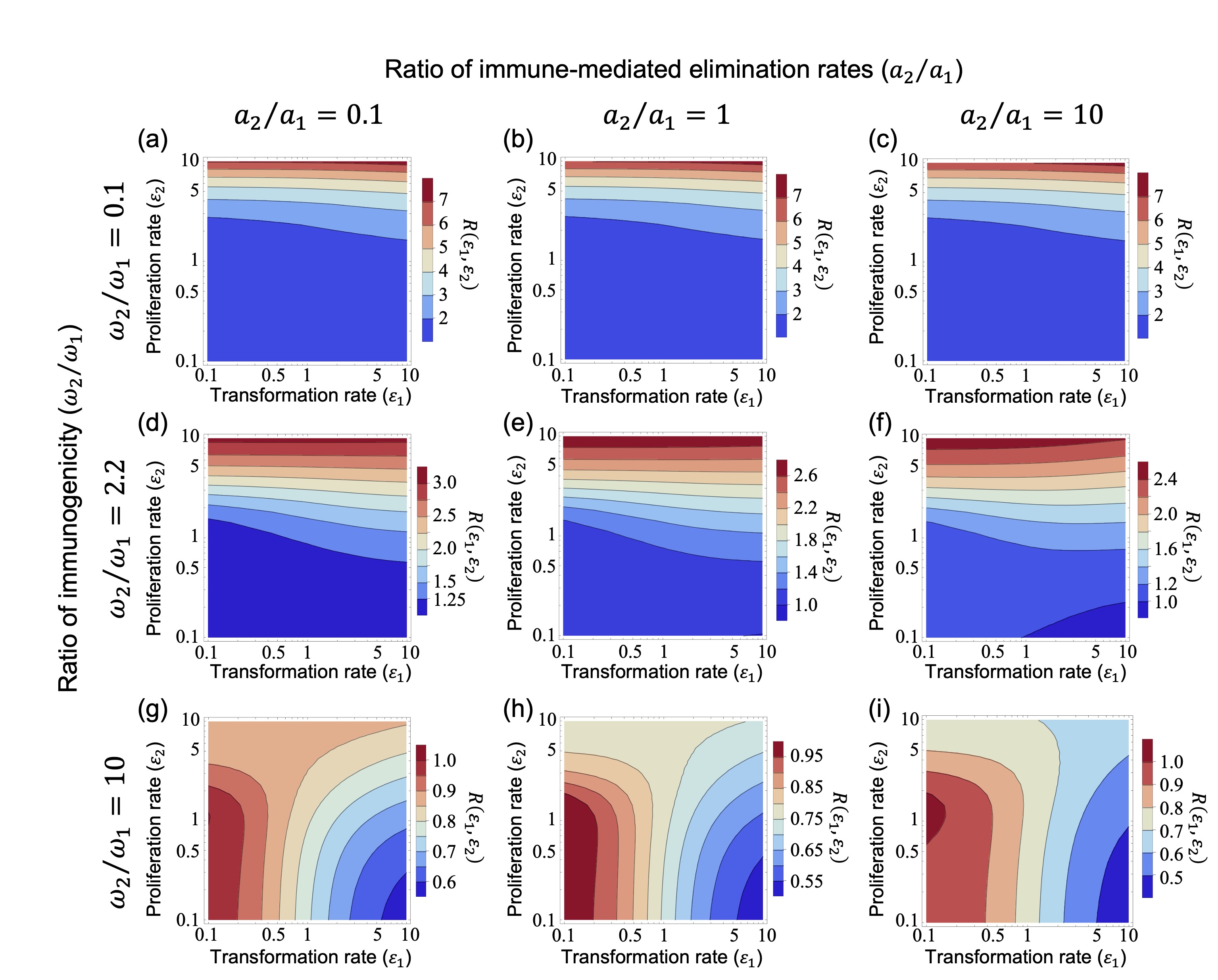
Figure S4-2. Effects of varying transformation and proliferation rates on the between-host reproduction number at the high additional mortality rate.** Contour maps showing the changes in the between-host reproduction number [$R\left( \varepsilon_{1}, \varepsilon_{2} \right)$] in response to various combinations of immunogenicity (${\omega_{2}}/{\omega_{1}}$) and immune-mediated elimination rates (${a_{2}}/{a_{1}}$) when the additional mortality rate was high ($m=5)$, plotted against transformation rates ($\varepsilon_{1}$, x-axis, log-scale) and proliferation rates ($\varepsilon_{2}$, y-axis, log-scale). From top to bottom, the rows increase the ratios of immunogenicity (${\omega_{2}}/{\omega_{1}}=0.1, 2.2, 10$), and the columns, from left to right, increase the ratios of immune-mediated elimination rates (${a_{2}}/{a_{1}}=0.1, 1, 10$), with fixed $\omega_{1}=0.001$, $a_{1}=0.01$, and the viral production ratio (${k_{2}}/{k_{1}}=0.1$). We set $b={2.09042\times10}^{-4}$ so that the between-host reproduction number of the virus without oncogenic effects is 1 [i.e. $R\left( 0,0 \right)=1$]. The colour transition from blue to red indicates increasing $R\left( \varepsilon_{1}, \varepsilon_{2} \right)$ values. (a)-(f) show an increase in $R\left( \varepsilon_{1}, \varepsilon_{2} \right)$ towards the upper-half region (= high proliferation area). In (g) and (h), higher $R\left( \varepsilon_{1}, \varepsilon_{2} \right)$ values are in the left-half region (= low transformation area). In contrast, (i) shows the optimal proliferation rates around $\varepsilon_{2}=1$, maximising $R\left( \varepsilon_{1}, \varepsilon_{2} \right)$.

**
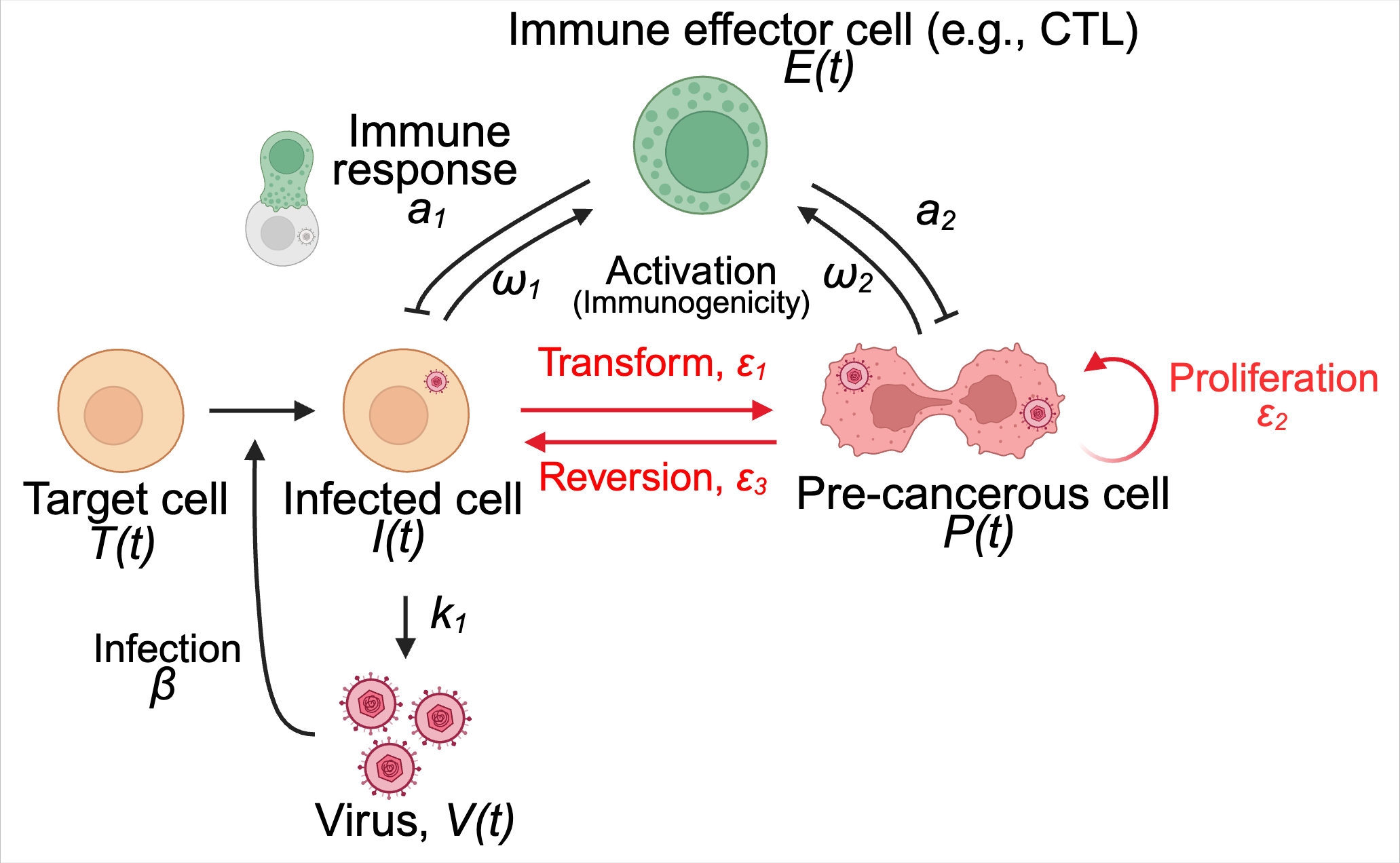
Figure S5. Schematic diagram of the different life cycle of oncogenic viruses such as KSHV and EBV.** In this model, pre-cancerous cells do not produce virions ($k_{2}=0$). When pre-cancerous cells are reactivated or re-enter the lytic phase, they return to the virion-producing cell class $I(t)$ at rate $\varepsilon_{3}$. This figure was created in BioRender. Koizumi, Y. (2025) https://BioRender.com/t40w913

**
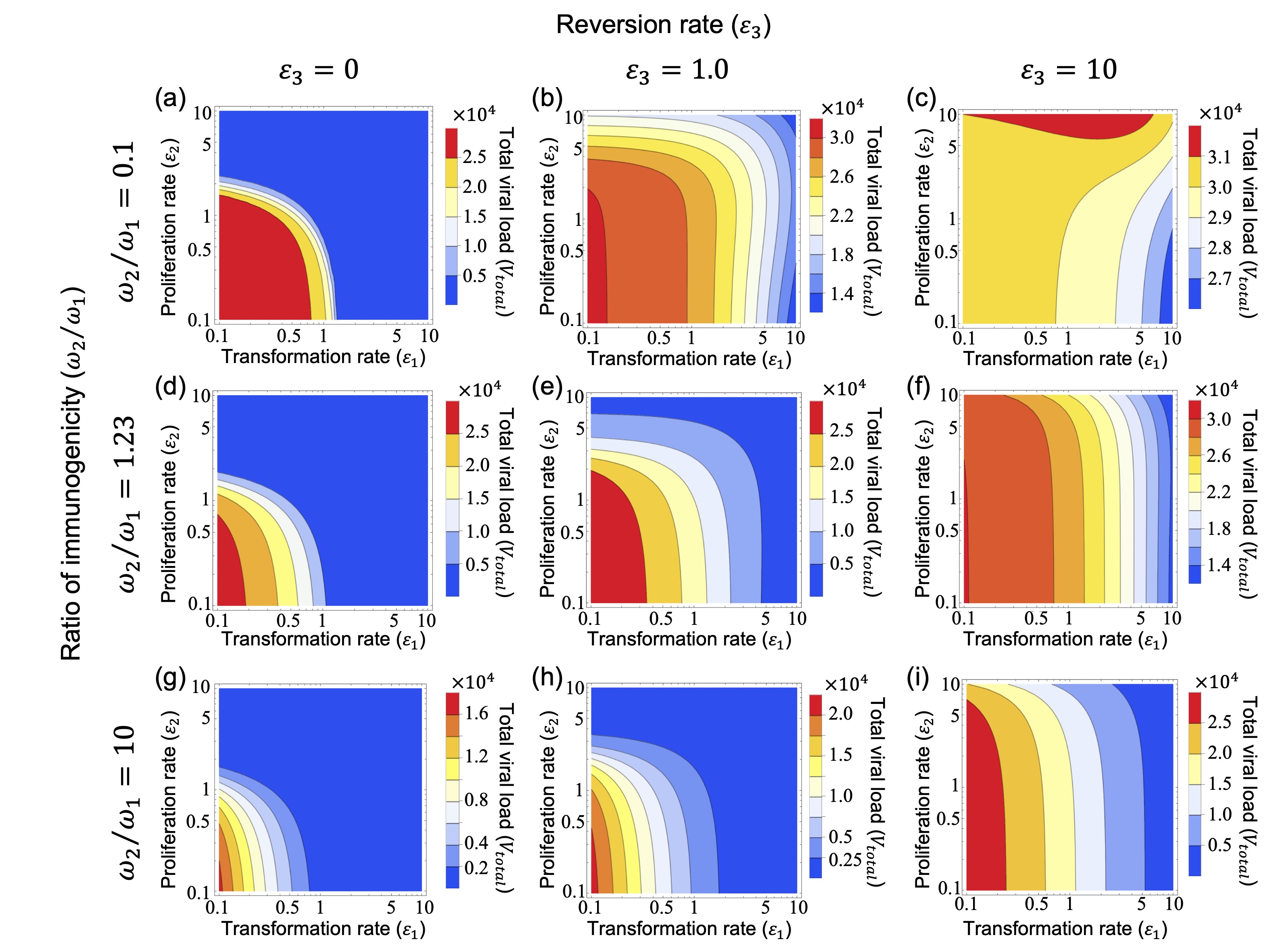
Figure S6-1. Effects of varying transformation and proliferation rates on the within-host total viral load in the different life cycle of oncogenic viruses.** Contour maps showing the changes in the within-host total viral load ($V_{total}$) in response to various combinations of immunogenicity (${\omega_{2}}/{\omega_{1}}$) and reversion rates ($\varepsilon_{3}$) when pre-cancerous cells do not produce virions ($k_{2}=0$), plotted against transformation rates ($\varepsilon_{1}$, x-axis, log-scale) and proliferation rates ($\varepsilon_{2}$, y-axis, log-scale). From top to bottom, the rows increase the ratios of immunogenicity (${\omega_{2}}/{\omega_{1}}=0.1, 1.23, 10$), and the columns, from left to right, increase the reversion rates ($\varepsilon_{3}=0, 1.0, 10$), with fixed $\omega_{1}=0.001$, $a_{1}=a_{2}=0.01$, and $k_{1}=0.1$. The colour transition from blue to red indicates increasing $V_{total}$ values. (a)-(c) show a transitional pattern in the fitness landscape at ${\omega_{2}}/{\omega_{1}}=0.1$. (a) and (b) show an increase in $V_{total}$ towards the lower-left corner. (c) shows optimal viral loads occur under high proliferation rates and intermediate transformation rates in the central upper area. In (d)-(i), when the immunogenicity of pre-cancerous cells is higher (${\omega_{2}}/{\omega_{1}}=1.23$ and $10$), higher $V_{total}$ values are in the lower-left corner.

**
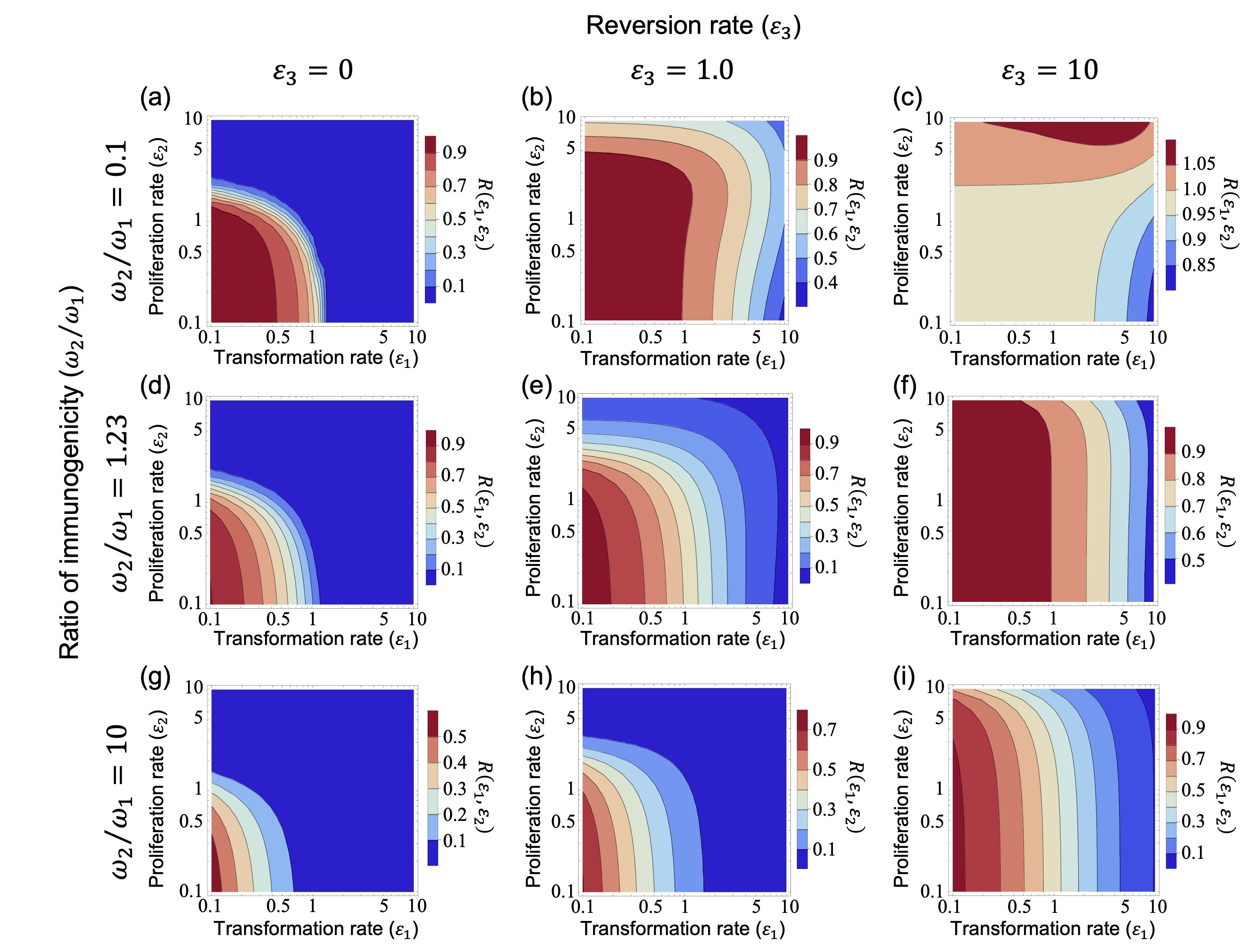
Figure S6-2. Effects of varying transformation and proliferation rates on the between-host reproduction number in the different life cycle of oncogenic viruses** **at the low additional mortality rate.** Contour maps showing the changes in the between-host reproduction number [$R\left( \varepsilon_{1}, \varepsilon_{2} \right)$] in response to various combinations of immunogenicity (${\omega_{2}}/{\omega_{1}}$) and reversion rates ($\varepsilon_{3}$) when pre-cancerous cells do not produce virions ($k_{2}=0$) and the additional mortality rate was low ($m=0.1)$, plotted against transformation rates ($\varepsilon_{1}$, x-axis, log-scale) and proliferation rates ($\varepsilon_{2}$, y-axis, log-scale). From top to bottom, the rows increase the ratios of immunogenicity (${\omega_{2}}/{\omega_{1}}=0.1, 1.23, 10$), and the columns, from left to right, increase the reversion rates ($\varepsilon_{3}=0, 1.0, 10$), with fixed $\omega_{1}=0.001$, $a_{1}=a_{2}=0.01$, and $k_{1}=0.1$. We set $b={3.5126\times10}^{-5}$ so that $R\left( 0,0 \right)=1$. The colour transition from blue to red indicates increasing $R\left( \varepsilon_{1}, \varepsilon_{2} \right)$ values. (a)-(c) show a transitional pattern in the fitness landscape at ${\omega_{2}}/{\omega_{1}}=0.1$. (a) and (b) show an increase in $R\left( \varepsilon_{1}, \varepsilon_{2} \right)$ towards the lower-left corner. (c) shows optimal $R\left( \varepsilon_{1}, \varepsilon_{2} \right)$ occur under high proliferation rates and intermediate transformation rates in the central upper area. In (d)-(i), when the immunogenicity of pre-cancerous cells is higher (${\omega_{2}}/{\omega_{1}}=1.23$ and $10$), higher $R\left( \varepsilon_{1}, \varepsilon_{2} \right)$ are in the lower-left corner. Note that the shapes of the fitness landscape of $R\left( \varepsilon_{1}, \varepsilon_{2} \right)$ are similar to that of $V_{total}$ (Fig. S6-1).

**
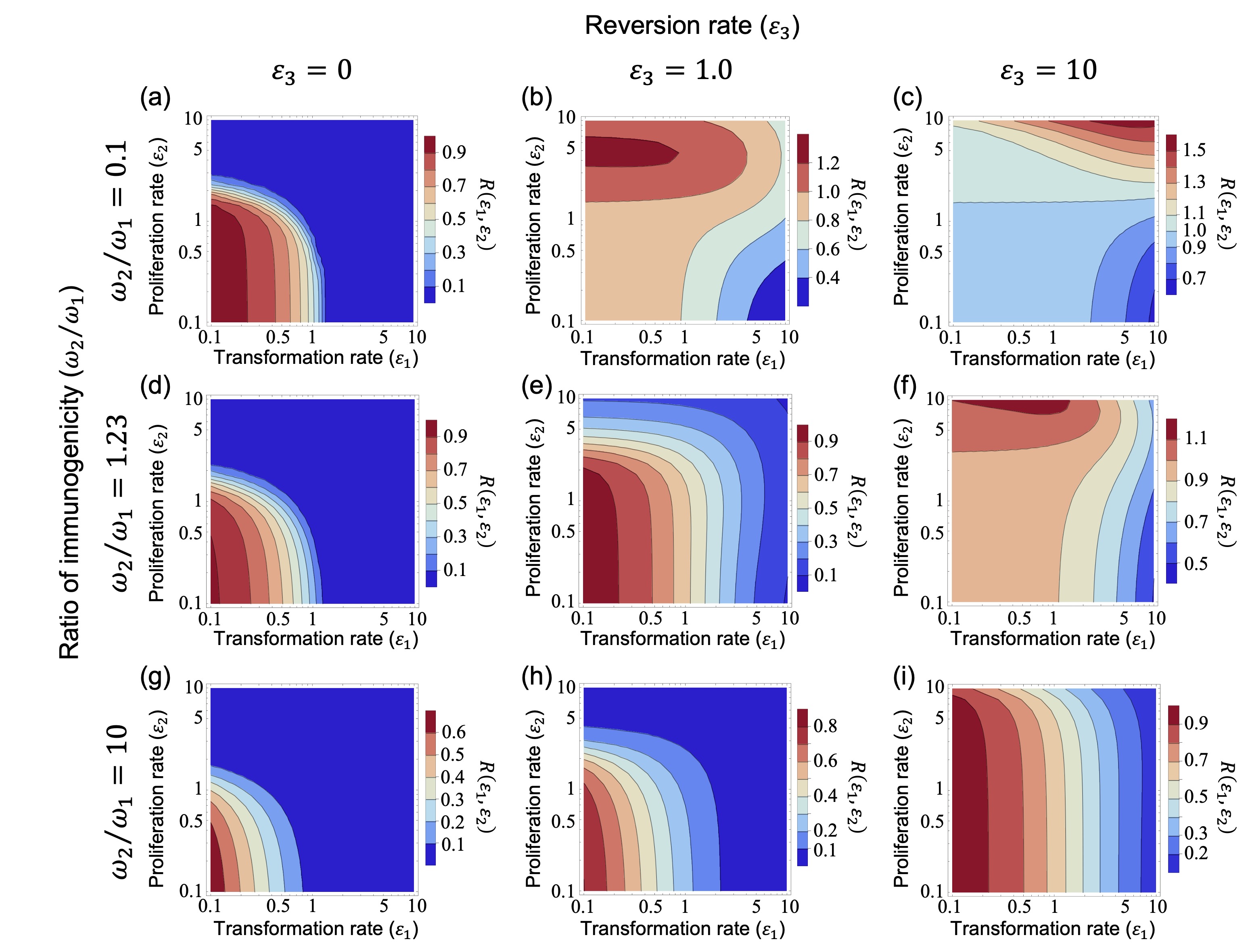
Figure S6-3. Effects of varying transformation and proliferation rates on the between-host reproduction number in the different life cycle of oncogenic viruses at the baseline additional mortality rate.** Contour maps showing the changes in the between-host reproduction number [$R\left( \varepsilon_{1}, \varepsilon_{2} \right)$] in response to various combinations of immunogenicity (${\omega_{2}}/{\omega_{1}}$) and reversion rates ($\varepsilon_{3}$) when pre-cancerous cells do not produce virions ($k_{2}=0$) and the additional mortality rate was the baseline value ($m=1.0)$, plotted against transformation rates ($\varepsilon_{1}$, x-axis, log-scale) and proliferation rates ($\varepsilon_{2}$, y-axis, log-scale). From top to bottom, the rows increase the ratios of immunogenicity (${\omega_{2}}/{\omega_{1}}=0.1, 1.23, 10$), and the columns, from left to right, increase the reversion rates ($\varepsilon_{3}=0, 1.0, 10$), with fixed $\omega_{1}=0.001$, $a_{1}=a_{2}=0.01$, and $k_{1}=0.1$. We set $b={5.865\times10}^{-5}$ so that $R\left( 0,0 \right)=1$. The colour transition from blue to red indicates increasing $R\left( \varepsilon_{1}, \varepsilon_{2} \right)$ values. (a)-(c) show a transitional pattern in the fitness landscape at ${\omega_{2}}/{\omega_{1}}=0.1$. (a) shows an increase in $R\left( \varepsilon_{1}, \varepsilon_{2} \right)$ towards the lower-left corner. (c) shows optimal $R\left( \varepsilon_{1}, \varepsilon_{2} \right)$ occur under high proliferation rates and transformation rates in the upper-right corner. Note that the shapes of the fitness landscape of $R\left( \varepsilon_{1}, \varepsilon_{2} \right)$ in (b) and (f) differ from those of $V_{total}$ (Fig. S6-1b&f) and the low-virulence case (Fig. S6-2b&f): $R\left( \varepsilon_{1}, \varepsilon_{2} \right)$ is maximised in the region with higher proliferation rates in (b) and (f).

**
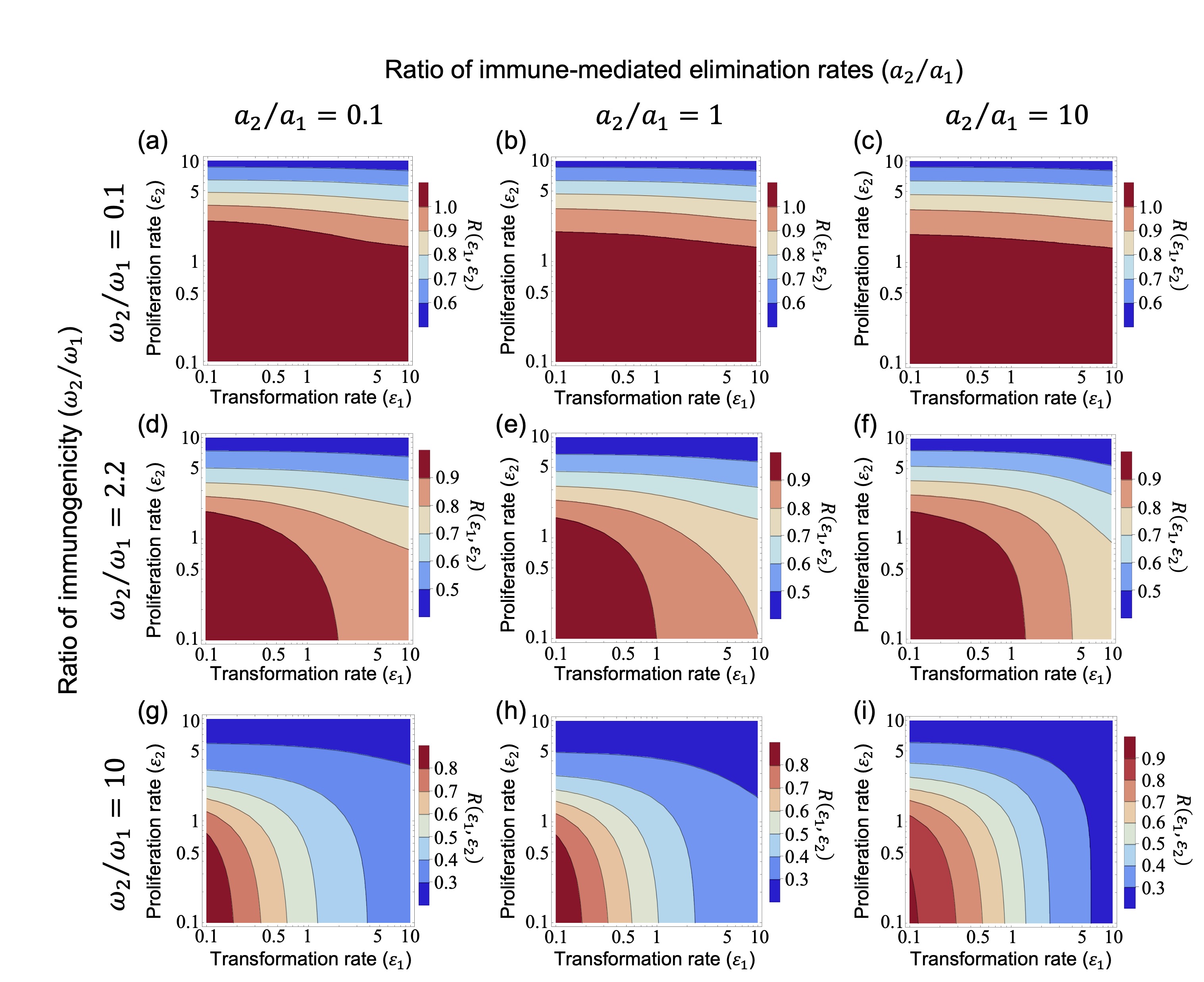
Figure S7-1. Effects of varying transformation and proliferation rates on the between-host reproduction number in a nonlinear relationship between viral load and host infectiousness (**$\boldsymbol{h=1}$**).** Contour maps showing the changes in the between-host reproduction number [$R\left( \varepsilon_{1}, \varepsilon_{2} \right)$] in response to various combinations of immunogenicity (${\omega_{2}}/{\omega_{1}}$) and immune-mediated elimination rates (${a_{2}}/{a_{1}}$) when the host infectiousness is the Hill function of viral load with $h=1$ [i.e. $B\left( t \right)=\beta_{BH\_max}{V\left( t \right)}/\left( V\left( t \right)+\beta_{50} \right)$]. From top to bottom, the rows increase the ratios of immunogenicity (${\omega_{2}}/{\omega_{1}}=0.1, 2.2, 10$), and the columns, from left to right, increase the immune-mediated elimination rates (${a_{2}}/{a_{1}}=0.1, 1, 10$), with fixed $\omega_{1}=0.001$, $a_{1}=0.01$, and the viral production ratio (${k_{2}}/{k_{1}}=1$). We set $b=\beta_{BH\_max}S_{0}=1.1818$ so that $R\left( 0,0 \right)=1$. The colour transition from blue to red indicates increasing $R\left( \varepsilon_{1}, \varepsilon_{2} \right)$ values. (a)-(c) show that $R\left( \varepsilon_{1}, \varepsilon_{2} \right)$ becomes lower in the region of high oncogenic effects (upper-right corner), which differs from the landscapes of $R\left( \varepsilon_{1}, \varepsilon_{2} \right)$ (Fig. 6). In (d)-(i), when the immunogenicity of pre-cancerous cells is higher (${\omega_{2}}/{\omega_{1}}=2.2$ and $10$), higher $R\left( \varepsilon_{1}, \varepsilon_{2} \right)$ are in the lower-left corner (= low oncogenicity).

**
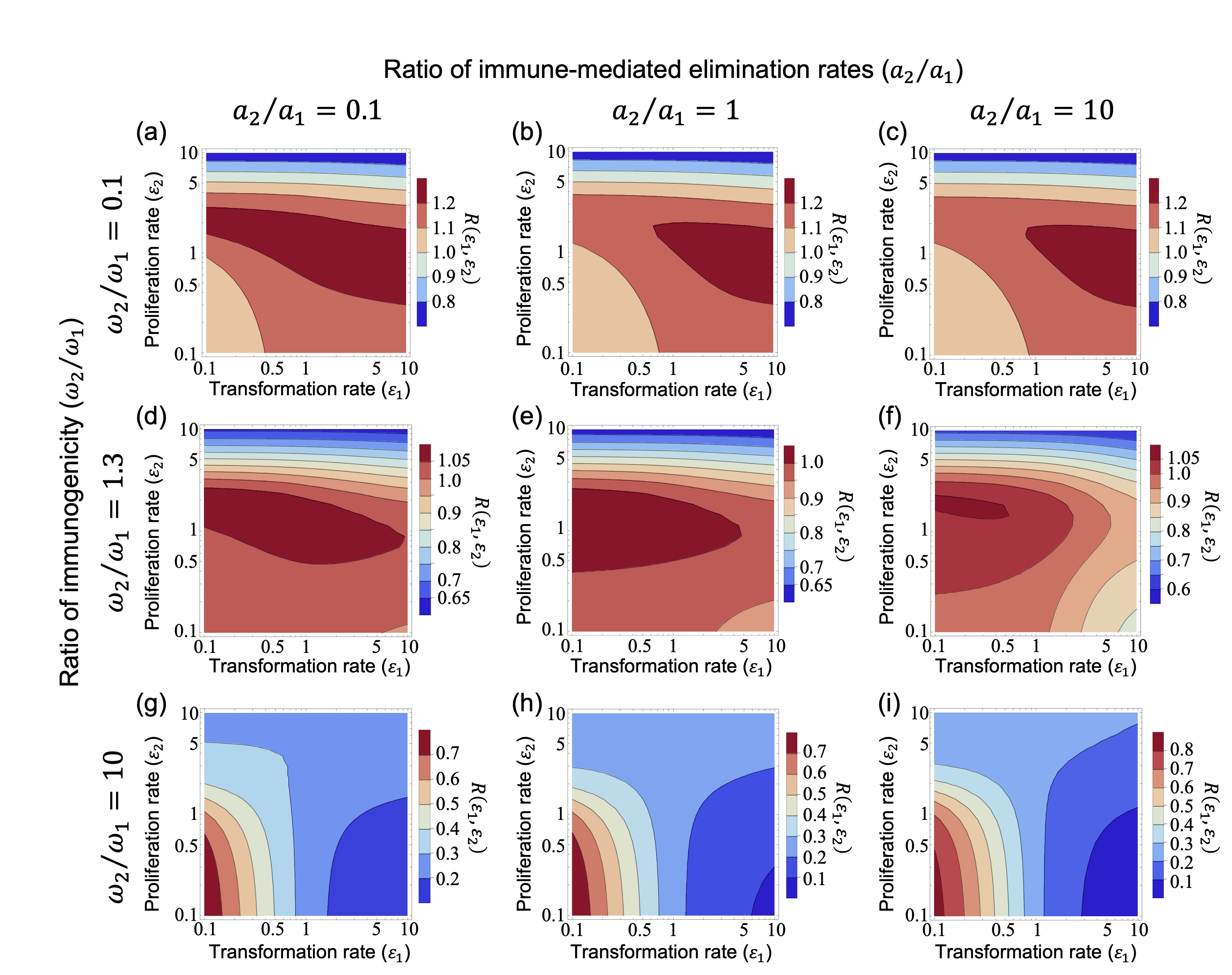
Figure S7-2. Effects of varying transformation and proliferation rates on the between-host reproduction number in a nonlinear relationship between viral load and host infectiousness (**$\boldsymbol{h=2}$**).** Contour maps showing the changes in the between-host reproduction number [$R\left( \varepsilon_{1}, \varepsilon_{2} \right)$] in response to various combinations of immunogenicity (${\omega_{2}}/{\omega_{1}}$) and immune-mediated elimination rates (${a_{2}}/{a_{1}}$) when the host infectiousness is the Hill function of viral load with $h=2$ [i.e. $B\left( t \right)=\beta_{BH\_max}{{V\left( t \right)}^{2}}/\left( {V\left( t \right)}^{2}+{\beta_{50}}^{2} \right)$]. From top to bottom, the rows increase the ratios of immunogenicity (${\omega_{2}}/{\omega_{1}}=0.1, 1.3, 10$), and the columns, from left to right, increase the immune-mediated elimination rates (${a_{2}}/{a_{1}}=0.1, 1, 10$), with fixed $\omega_{1}=0.001$, $a_{1}=0.01$, and the viral production ratio (${k_{2}}/{k_{1}}=1$). We set $b=\beta_{BH\_max}S_{0}=1.5483$ so that $R\left( 0,0 \right)=1$. The colour transition from blue to red indicates increasing $R\left( \varepsilon_{1}, \varepsilon_{2} \right)$ values. (a)-(f) show that there is an optimal region in the intermediate proliferation rates that maximises $R\left( \varepsilon_{1}, \varepsilon_{2} \right)$. In (g)-(i), when the immunogenicity of pre-cancerous cells is higher (${\omega_{2}}/{\omega_{1}}=10$), higher $R\left( \varepsilon_{1}, \varepsilon_{2} \right)$ are in the lower-left corner (= low oncogenicity).

**
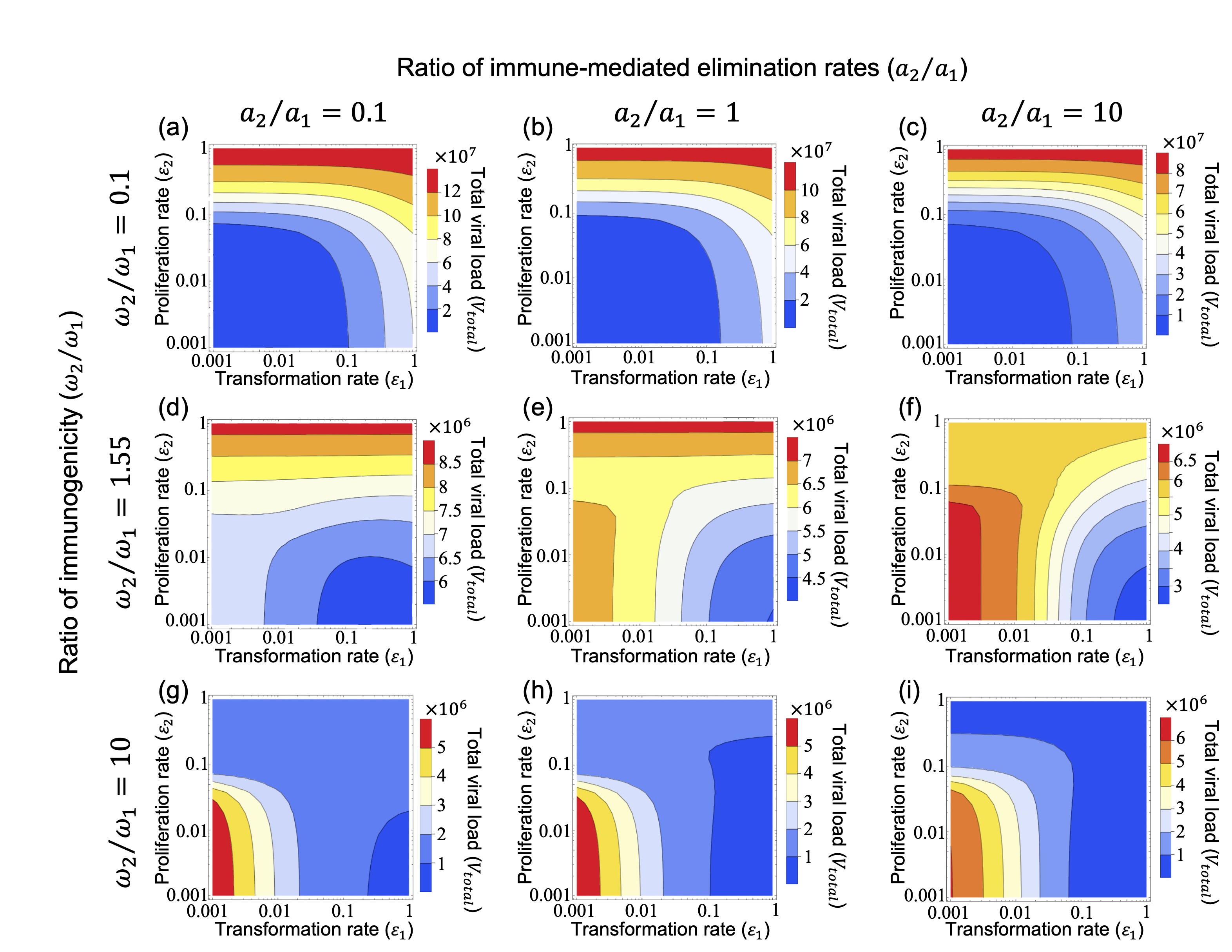
Figure S8-1. Effects of different transformation and proliferation rates on the within-host total viral load in a HPV-specific model.** Contour maps showing the changes in within-host total viral load ($V_{total}$) in response to various combinations of immunogenicity (${\omega_{2}}/{\omega_{1}}$) and immune-mediated elimination rates (${a_{2}}/{a_{1}}$), plotted against transformation rates ($\varepsilon_{1}$, x-axis, log-scale) and proliferation rates ($\varepsilon_{2}$, y-axis, log-scale). From top to bottom, the rows increase the ratios of immunogenicity (${\omega_{2}}/{\omega_{1}}=0.1, 1.55, 10$), and the columns, from left to right, increase the ratios of immune-mediated elimination rates (${a_{2}}/{a_{1}}=0.1, 1, 10$), with fixed $\omega_{1}=0.001$, $a_{1}=0.01$, and the viral production ratio (${k_{2}}/{k_{1}}=1$). The colour transition from blue to red indicates increasing $V_{total}$ values. (a)-(c) show an increase in $V_{total}$ towards the upper-right corner at ${\omega_{2}}/{\omega_{1}}=0.1$; (g)-(i) show an opposite trend with $V_{total}$ increasing towards the lower-left corner at ${\omega_{2}}/{\omega_{1}}=10$. (d)-(f) show a transitional pattern at ${\omega_{2}}/{\omega_{1}}=1.55$, where the region of higher $V_{total}$ shifts from the upper-half region (= high proliferation area) to the lower-left corner (= low oncogenicity area).

**
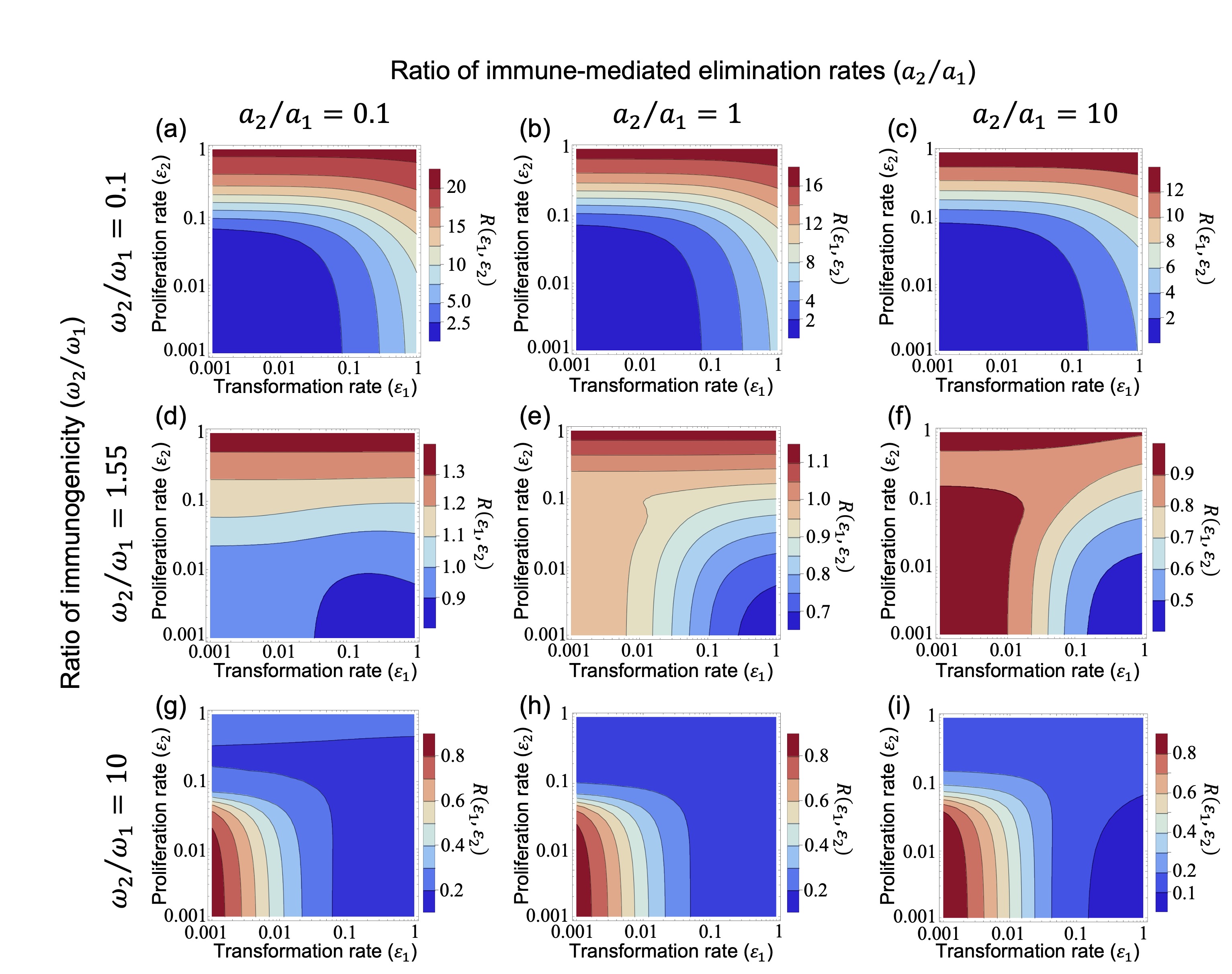
Figure S8-2. Effects of different transformation and proliferation rates on the between-host reproduction number in a HPV-specific model.** Contour maps showing the changes in $R\left( \varepsilon_{1}, \varepsilon_{2} \right)$ in response to various combinations of immunogenicity (${\omega_{2}}/{\omega_{1}}$) and immune-mediated elimination rates (${a_{2}}/{a_{1}}$), plotted against transformation rates ($\varepsilon_{1}$, x-axis, log-scale) and proliferation rates ($\varepsilon_{2}$, y-axis, log-scale). From top to bottom, the rows increase the ratios of immunogenicity (${\omega_{2}}/{\omega_{1}}=0.1, 1.55, 10$), and the columns, from left to right, increase the ratios of immune-mediated elimination rates (${a_{2}}/{a_{1}}=0.1, 1, 10$), with fixed $\omega_{1}=0.001$, $a_{1}=0.01$, and the viral production ratio (${k_{2}}/{k_{1}}=1$). We fixed $\mu={10}^{-4}$ and set $b=\beta_{BH}S_{0}=1.56792\times{10}^{-7}$ so that $R\left( 0,0 \right)=1$. The colour transition from blue to red indicates increasing $R\left( \varepsilon_{1},\varepsilon_{2} \right)$ values. (a)-(c) show an increase in $R\left( \varepsilon_{1},\varepsilon_{2} \right)$ towards the upper-right corner at ${\omega_{2}}/{\omega_{1}}=0.1$; (g)-(i) show an opposite trend with $R\left( \varepsilon_{1},\varepsilon_{2} \right)$ increasing towards the lower-left corner at ${\omega_{2}}/{\omega_{1}}=10$. (d)-(f) show a transitional pattern at ${\omega_{2}}/{\omega_{1}}=1.55$, where the region of higher $R\left( \varepsilon_{1},\varepsilon_{2} \right)$ shifts from the upper-half region (= high proliferation) to the lower-left corner (= low oncogenicity). Note that (f) shows a distinctive dual-peak pattern in $R\left( \varepsilon_{1},\varepsilon_{2} \right)$, located in the upper and lower-left regions.

**
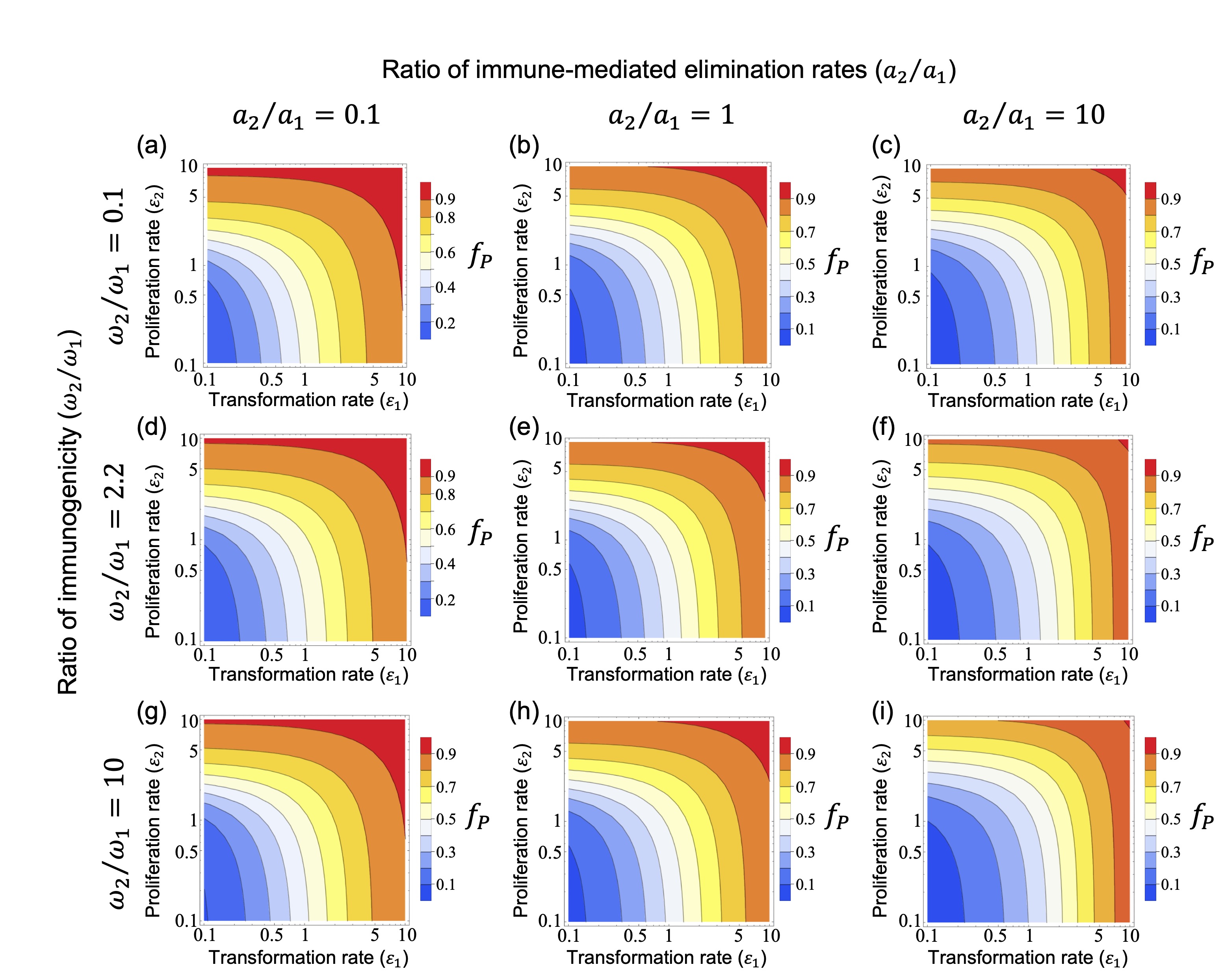
Figure S9. Effects of different transformation and proliferation rates on the proportion of total viral production contributed by pre-cancerous cells.** Contour maps showing the changes in the proportion of total viral production from pre-cancerous cells ($f_{p}$) in response to various combinations of immunogenicity (${\omega_{2}}/{\omega_{1}}$) and immune-mediated elimination rates (${a_{2}}/{a_{1}}$), plotted against transformation rates ($\varepsilon_{1}$, x-axis, log-scale) and proliferation rates ($\varepsilon_{2}$, y-axis, log-scale). From top to bottom, the rows increase the ratios of immunogenicity (${\omega_{2}}/{\omega_{1}}=0.1, 2.2, 10$), and the columns, from left to right, increase the ratios of immune-mediated elimination rates (${a_{2}}/{a_{1}}=0.1, 1, 10$), with fixed $\omega_{1}=0.001$, $a_{1}=0.01$, and the viral production ratio (${k_{2}}/{k_{1}}=1$). The colour transition from blue to red indicates increasing $f_{p}$ values. (a)-(i) show an increase in $f_{p}$ towards the upper-right corner (= high oncogenicity), regardless of the values of ${\omega_{2}}/{\omega_{1}}$ and ${a_{2}}/{a_{1}}$.

**References**

Fraser, C., et al. (2007), 'Variation in HIV-1 set-point viral load: epidemiological analysis and an evolutionary hypothesis', *Proceedings of the National Academy of Sciences of the United States of America,* 104 (44), 17441-6.

Ke, R., et al. (2021), 'In vivo kinetics of SARS-CoV-2 infection and its relationship with a person's infectiousness', *Proceedings of the National Academy of Sciences of the United States of America,* 118 (49), e2111477118.

Murall, C. L., Bauch, C. T., and Day, T. (2015), 'Could the human papillomavirus vaccines drive virulence evolution?', *Proceedings: Biological Sciences,* 282 (1798), 20141069.
